# Cationic copolymers that enhance wild-type-specific suppression in BNA-clamp PCR and preferentially increase the *T*_m_ of fully matched complementary DNA and BNA strands

**DOI:** 10.1093/biomethods/bpac009

**Published:** 2022-03-30

**Authors:** Ami Tachibana, Nahohiro Fujimura, Minoru Takeuchi, Koji Watanabe, Yoko Teruuchi, Tomoaki Uchiki

**Affiliations:** 1 Nitto Boseki Co. Ltd, Kawasaki, Kanagawa 210-0821, Japan; 2 Nittobo Medical Co. Ltd, Koriyama, Fukushima 963-8061, Japan

**Keywords:** bridged nucleic acid, PCR clamping, cationic copolymers, rare allele enrichment, DNA melting temperature, mutant detection

## Abstract

Mutation detection is of major interest in molecular diagnostics, especially in the field of oncology. However, detection can be challenging as mutant alleles often coexist with excess copies of wild-type alleles. Bridged nucleic acid (BNA)-clamp PCR circumvents this challenge by preferentially suppressing the amplification of wild-type alleles and enriching rare mutant alleles. In this study, we screened cationic copolymers containing nonionic and anionic repeat units for their ability to (i) increase the *T_m_* of double-stranded DNA, (ii) avoid PCR inhibition, and (iii) enhance the suppression of wild-type amplification in BNA-clamp PCR to detect the KRAS G13D mutation. The selected copolymers that met these criteria consisted of four types of amines and anionic and/or nonionic units. In BNA-clamp PCR, these copolymers increased the threshold cycle (*C*_t_) of the wild-type allele only and enabled mutation detection from templates with a 0.01% mutant-to-wild-type ratio. Melting curve analysis with 11-mer DNA–DNA or BNA–DNA complementary strands showed that these copolymers preferentially increased the *T_m_* of perfectly matched strands over strands containing 1-bp mismatches. These results suggested that these copolymers preferentially stabilize perfectly matched DNA and BNA strands and thereby enhance rare mutant detection in BNA-clamp PCR.

## Introduction

Mutant allele detection is of major interest in biology and medicine. Molecular diagnostics for mutant detection is becoming a critical tool to decide treatment regimens, especially in oncology [1, 2]. However, as a mutation usually arises from a large population of wild-type alleles, mutant alleles are often buried in an excess of wild-type copies. This poses a challenge for their detection [3, 4]. These rare mutant alleles can potentially be identified by amplification of the target gene fragments followed by various detection techniques including Sanger sequencing [5–7], pyrosequencing [8], next-generation sequencing (NGS) [9], matrix assisted laser desorption/Ionization time of flight mass spectrometry (MALDI-TOF MS) [10], Taqman probes [7], denaturing high performance liquid chromtography (HPLC) [11], and melting curve analysis [12, 13]. Nevertheless, with these detection techniques alone, sensitivity for the mutant-to-wild-type ratio is often limited to 5–25% [4–8, 10, 13–15]. As cancer-derived mutant alleles are often buried in a much larger population of wild-type alleles, higher sensitivity is desirable to provide patients with unequivocal diagnoses [4]. To overcome this limitation, numerous enrichment techniques have been developed to preferentially amplify mutant alleles or eliminate wild-type alleles [3, 4, 14, 15]. Currently, these mutant enrichment and detection techniques are often combined to achieve high sensitivity for the detection of alleles in low abundance [3, 14–18].

A common strategy for mutant enrichment is allele-specific PCR (AS PCR) that utilizes allele-specific primers containing mismatches toward the 3′-ends [3, 7, 19]. By optimizing annealing conditions, AS PCR achieves mutant allele enrichment by 100- to 1000-fold [4, 15]. Another strategy is to eliminate wild-type alleles using thermo-stable restriction enzymes that specifically digest them [20]. A different use of restriction enzymes is to digest mutant alleles first and then ligate exogenous oligonucleotide tags in the ends of the digested fragments from which mutant alleles can be amplified [21]. The use of restriction enzymes can achieve 10^3^–10^6^-fold enrichment of mutant alleles [4]. An alternative strategy is to suppress amplification of the wild-type allele by complimentary strands that preferentially maintain the double-stranded structure of wild-type alleles but not the mutant allele during PCR. COLD PCR employs such a strategy using entire stretches of amplicon as the wild-type blocking complementary strand and it can enrich mutant alleles by 5- to 100-fold [4, 17]. In recent years, the use of shorter complementary strands, generally called nucleic acid clamps, is becoming a major approach for enrichment of low abundant alleles [22, 23]. This approach achieves mutant allele enrichment by 100- to 1000-fold [4, 14, 24, 25]. In addition to the high degree of enrichment, nucleic acid clamps are cost-effective and relatively easy to design. They are also compatible with various sequencing platforms [14] and amplification strategies, including isothermal methods and asymmetric PCR [24, 26].

A nucleic acid clamp is a stretch of nucleic acid analogs that form more stable base pairs when compared with natural DNA. The increased stability also creates larger differences in the melting temperatures between strands with base-pair mismatches and perfect matches, leading to higher specificity. Peptide nucleic acid (PNA) [27] and bridged nucleic acid (BNA) [28] are widely used building blocks of nucleic acid clamps incorporated in various rare allele detection systems [22, 24, 26, 29–31]. PNA consists of purine and pyrimidine bases attached to the side chain of an amide-linked *N*-(2-amino-ethyl)-glycine peptide backbone [24]. It is chemically stable and resistant to the exonuclease activity of DNA polymerase and is suitable as a clamp to suppress the amplification of wild-type alleles. A PNA clamp blocks strand elongation not only when it binds in the middle of the target fragment but also the primer annealing site, and it thus cannot be used as a primer. BNA consists of nucleotides with sugars locked in the 3′-endo conformation by a bridge between the ribose 2′ oxygen and the 4′ carbon atoms [3, 32]. It also increases the melting temperature and specificity of hybridization [3, 32]. Unlike PNA, BNA can serve as a primer [33] and be incorporated into DNA, allowing flexible adjustment of melting temperatures and the avoidance of stem loop formation within the clamp [34]. Nevertheless, the original version of BNA, which has a methylene bridge, is susceptible to 3′–5′ exonuclease DNA polymerase activity, and this degradation limits its use as a clamp [32]. Therefore, instead of mutant enrichment, it is often integrated into detection probes in real-time PCR to specifically bind to a target allele sequence and emit fluorescence signals when cleaved by the exonuclease activity during PCR [3, 14, 30]. The initial version of BNA has also been synthesized as a locked nucleic acid (LNA) [35, 36] and it is often combined with PNA as a pair consisting of a mutant-specific probe and wild-type blocking clamp [3, 14, 24, 26, 37].

In the development of a new generation of BNA, the 2′ oxygene and the 4′ carbon bridge were modified to include additional non-methylene groups that make it resistant to exonuclease activity [22, 32, 38] and allow for modulation of the hybridization stability and selectivity [22, 32]. Among them, analogs with a bridge containing amine (hereafter, 2′4′-BNA(NC)) are suitable as a building block for nucleotide clamps and are currently used in several systems to preferentially amplify rare mutant alleles, especially for cancer diagnosis [39–41]. Nevertheless, only a few BNA-clamp PCR commercial products are available and their clinical use is limited [39]. For more robust use of BNA-clamp PCR, higher sensitivity of mutant detection may be desirable, as it is possible for other more commonly used methods to give a sensitivity of a 0.01% or lower mutant-to-wild-type ratio [3, 4].

In this study, we explored the use of cationic copolymers that incorporate nonionic and anionic repeat units in order to further enhance the detection limit of BNA-clamp PCR. Previous studies have shown that cationic copolymers consisting of poly-lysine and graft-dextran stabilize dsDNA [42, 43] and enhance the discrimination of perfectly matched and mismatched complementary DNA strands in assays driven by DNA strand exchange [44–46]. As the same copolymers also show synergic stabilization of triplex oligonucleotides with BNA [47], we thought that other copolymers might also give synergic effects in BNA-clamp PCR. To test this, we screened a series of copolymers and identified a few that enhanced wild-type-specific suppression in BNA-clamp PCR, which further decreased the mutant-to-wild-type ratio detection limit. These polymers also showed preferential increases of *T_m_* in complementary DNA–DNA and BNA–DNA strands, suggesting their potential application to other systems. As far as we know, this is the first study to demonstrate improved sensitivity of BNA-clamp PCR by the addition of polymers. These copolymers may promote clinical applications of BNA-clamp PCR and give it a competitive edge over other methods for low-abundance allele detection.

## Materials and methods

### Polymers

The following polymers were purchased from Nittobo Medical (Fukuyama, Japan) (see Supplementary Table S2): (i) homo- and copolymers of allylamine hydrochloride; (ii) homo- and copolymers of diallylamine hydrochloride, with the exception of the ternary copolymer containing acrylamide and acrylic acid repeat units (P18); and (iii) homo- and co-polymers of diallylmethylamine, with the exception of the binary copolymer containing acrylamide repeat units (P21). These two exceptions of copolymers were specifically synthesized for this study. Both homo- and copolymers of {*N*-[3-(dimethylamino)propyl](meth)acrylamide} hydrochloride as well as polyacrylamide and polyacrylic acid were synthesized specifically for this study.

All polymers were synthesized from mixtures of monomers at ratios specified in Supplementary Table S2. These monomers were first weighed to yield a specific molarity (0.1–20 moles) and placed in a glass flask with a stirrer, a thermometer, and a cooling unit. Distilled water was added to the mixture of the monomers and heated to 55–65°C. After polymerization, the products (i.e. polymers) were precipitated by the addition of optimum organic solvents and purified. The product formation was evaluated based on the yields of the precipitated products and/or formation and recovery of high molecular weight materials by gel permeation chromatography. For the polymers that were not produced by Nittobo Medical, detailed procedures for their synthesis and analysis of the product formation are described in Supplementary Materials and Supplementary Table S4, respectively.

### Synthetic oligonucleotides

In this study, KRAS and BRAF genes were used as model systems and several oligonucleotides were synthesized for different purposes: 40-mer and 11-mer dsDNA for melting curve analysis, ssDNA as primers for PCR, and 11-mer single-strand BNA complementary to the site of KRAS G13. These are listed in Table 1.

BNA clamp and PCR primers were included in the BNA® Clamp PCR Enrichment Kit KRAS (Riken Genesis Inc.) & BNA® Clamp PCR Enrichment Kit BRAF (Riken Genesis Inc.) The sequences of the primers and BNA-clamp included in the KRAS clamp PCR kit and the BRAF clamp PCR kit are not disclosed by the manufacturer. Nevertheless, for the KRAS clamp PCR kit, we deduced the sequences of amplification primers and BNA-clamp from a corresponding patent publication [48] (Table 1). Nearly all other oligonucleotides were custom-synthesized by Fasmac Inc. (except for KRAS-S antisense wt-B, which was custom-synthesized by GeneDesign Inc.). Sense and antisense oligonucleotides of equivalent length were mixed together as complementary strands for the melting curve analysis.

**Table 1.** Oligonucleotides used in this study

Name	Sequence (5′ → 3′)	Legth (bp)	Nucleotide type	Purpose	Supplier
KRAS-L sense wt	GTGGTAGTTGGAGCTGGTGGCGTAGGCAAGAGTGCCTTGA	40	DNA	Melting curve analysis	Fasmac Inc.
KRAS-L antisense wt	TCAAGGCACTCTTGCCTACGCCACCAGCTCCAACTACCAC	40	DNA	Melting curve analysis	Fasmac Inc.
KRAS-S sense wt	GGTGGCGTAGG	11	DNA	Melting curve analysis	Fasmac Inc.
KRAS-S sense mt	GGTGACGTAGG	11	DNA	Melting curve analysis	Fasmac Inc.
KRAS-S antisense wt	CCTACGCCACC	11	DNA	Melting curve analysis	Fasmac Inc.
KRAS-S antisense wt-B	cctAcGccAcc	11	BNA+DNA	Melting curve analysis	GeneDesign Inc.
KRAS forward primer	GCCTGCTGAAAATGACTGAATATA	24	DNA	Real-time PCR	Fasmac Inc.
KRAS reverse primer	CAAGATTTACCTCTATTGTTGGA	23	DNA	Real-time PCR	Fasmac Inc.
KRAS kit forward primer	CTGAATATAAACTTGTGGTAGTTGG	25	DNA	BNA clamp PCR	Riken Genesis Inc.
KRAS kit reverse primer	GTCCTGCACCAGTAATATGC	20	DNA	BNA clamp PCR	Riken Genesis Inc.
KRAS kit BNA clamp	cctAcGccAcc	11	BNA+DNA	BNA clamp PCR	Riken Genesis Inc.

BNA bases are indicated in lower case. Sequences of the primers included in BNA^®^ Clamp PCR Enrichment Kit KRAS (“KRAS kit forward primer,” “KRAS kit reverse primer,” and “KRAS kit BNA clamp” supplied by Riken Genesis Inc.) were deduced from a corresponding patent publication [48]. Sequences of amplification primers for BNA^®^ Clamp PCR Enrichment Kit BRAF are not disclosed. Sequences of sequencing primers for both BNA^®^ Clamp PCR Enrichment Kit KRAS and BRAF are not disclosed.

### Template DNA for PCR

For the majority of experiments involving PCR, genomic DNA from cultured cells was used as a template. HCC70 and MDA-MB-231 human breast cancer cell lines were used as sources of KRAS wild-type and G13D mutant, respectively. HCC70 and DU4475 human breast cancer cell lines were used for BRAF wild-type and V600E, respectively. These cells were initially purchased from ATCC. HCC70 and DU4475 cells were cultured in RPMI1640 medium (Gibco Inc.), and MDA-MB-231 cells were cultured in Leibovitz’s L15 medium (Gibco Inc.). Their genomic DNA was extracted using a QIAamp DNA Mini Kit (QIAGEN Inc.). Plasmids containing KRAS wild-type and G13D mutant genes were custom synthesized by Fasmac Inc. for the remaining experiments.

### Melting curve analysis

Melting curve analysis was performed on a StepOnePlus Real Time PCR system (Applied Biosystems Inc.) in a 20-µL reaction mixture containing 1.25 µM EvaGreen dye (Biotium Inc.) and 42.5 µM of 40-mer complementary DNA–DNA strands, 100 µM 11-mer complementary DNA–DNA, or 50 µM DNA–BNA strands (Table 1) and 1.0% (w/v) cationic polymers (Tables 2 and 3) in deionized water (polymer concentration was set at 1.0% (w/v) as most of these polymers were supplied at 10% w/v), and 10-fold dilution was convenient). The samples were initially heated at 95°C to allow denaturation. For the 40-mer strands, samples were cooled down to 40°C and held at 40°C for 1 min to allow for renaturation and intercalation of the dye. For the 11-mer strands, samples were cooled down to 10°C and held at 10°C for 1 min instead. Samples were then heated up to 95°C to allow for denaturation and held at 95°C for 15 s. During denaturation, fluorescence signals were recorded at every 0.3°C increment. Melting curves were analyzed by StepOne™ Software v2.3 accompanied with the instrument above (Applied Biosystems Inc.). The derivative of fluorescence intensity (−d(fluor. intensity)/d(temperature)) was plotted against temperature and temperature at the peak maximum was taken as the melting temperature (*T*_m_).

**Table 2.** Effects of poly(allylamine hydrochloride) (P(AA[HCl])) and poly{*N*-[3-(dimethylamino)propyl](meth)acrylamide}hydrochloride (P(DMAPMA[HCl])) on *T*_m_ and PCR

Poymer number	Types of repeat units	Ratio of repeat units^a^	*T*_m_ (°C)	Degree of *T*_m_ increase (°C)	PCR
P1	P(AA[HCl])	Homopolymer	Could not be detected	N/A	Complete inhibition
P2	P(AA[HCl]/MA)	N/Ds	82.93	23.93	No inhibition
P3	P(DMAPMA[HCl])	Homopolymer	Could not be detected	N/A	Complete inhibition
P4	P(DMAPMA[HCl]/AAm)	3:1	Could not be detected	N/A	Complete inhibition
P5		1:1	Could not be detected	N/A	Complete inhibition
P6		1:3	Could not be detected	N/A	Complete inhibition
P7	P(DMAPMA[HCl]/AAc)	3:1	91.55	35.93	Complete inhibition
P8		1:1	84.07	28.45	Slight inhibition
P9		1:3	78.68	23.06	No inhibition
P10	P(DMAPMA[HCl]/AAm/AAc)	1:1:1	86.52	28.27	Slight inhibition
P11		2:1:1	89.96	31.71	Complete inhibition
P12		1:1:2	79.64	21.39	No inhibition
P13	P(AAm) (control)	Homopolymer	66.03	5.54	No inhibition
P14	P(AAc) (control)	Homopolymer	75.45	14.96	Medium level inhibition ^b^
Chemical structure of the repeat units: Each structure is labeled with full name of the corresponding polymers followed by their abbreviation in bracket.					
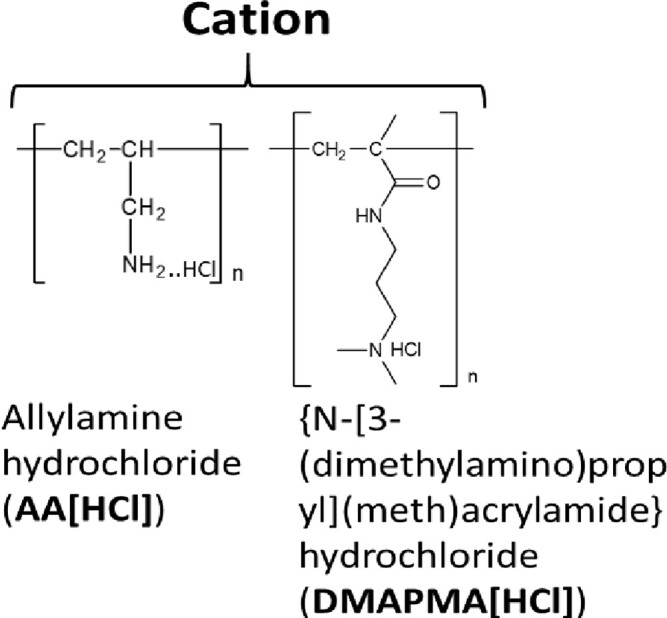		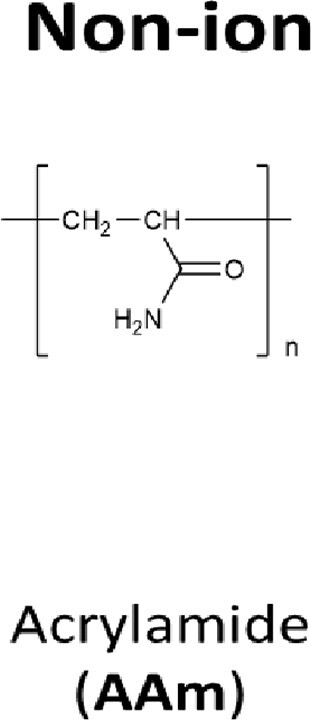		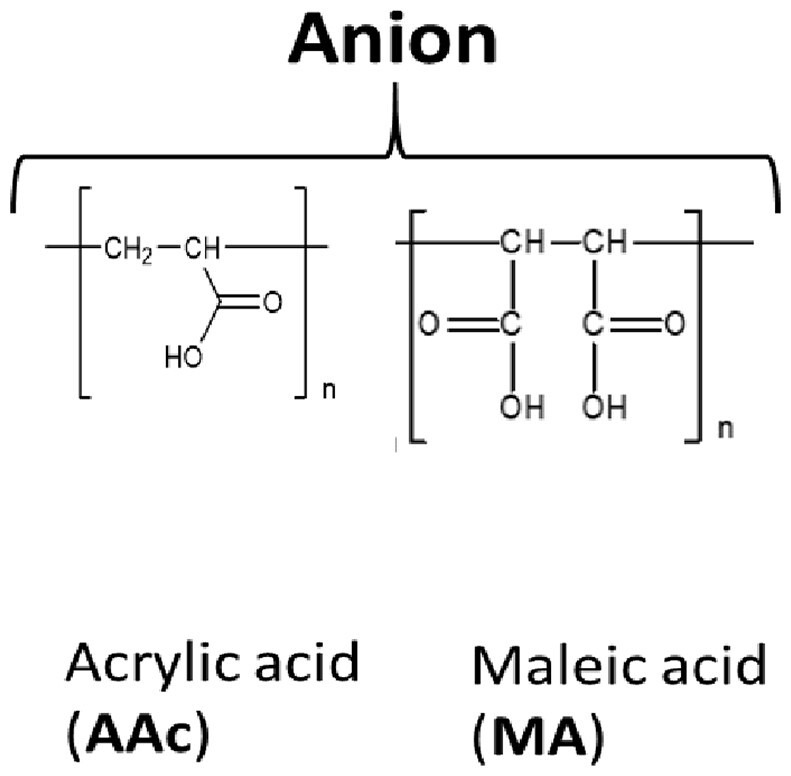	

a Ratio of monomers used in synthesis.

b Addition of P14 caused levels of inhibition higher than those labeled “slightly inhibited,” but increases of the signal corresponding to the amplification was still observed. N/Ds, not disclosed by the supplier. N/A, not applicable as dsDNA formation was apparently disrupted. For the melting curve analysis, all the polymers’ concentrations were 1% (w/v). For the real-time PCR, all the polymers’ concentrations were 0.01% (w/v). *T*_m_ without polymer (reference temperature) was 58°C. *T*_m_ with 1.5 mM MgCl_2_ was 80°C.

**Table 3.** Effects of poly(diallylamine hydrochloride) (P(DAA[HCl])) and poly(diallylmethylamine hydrochloride) (P(DAMA[HCl])) on *T*_m_ and PCR

Poymer number	Types of repeat units	Ratio of repeat units^a^	*T*_m_ (°C)	Degree of *T*_m_ increase (°C)	PCR
P15	P(DAA[HCl])	Homopolymer	79.94	20.94	Complete inhibition
P16	P(DAA[HCl]/SO_2_ )	1:1	78.14	19.14	Complete inhibition
P17	P(DAA[HCl]/MA)	N/Ds	83.23	24.23	No inhibition
P18	P(DAA[HCl]/AAm/AAc)	1:1:2	79.04	20.79	No inhibition
P19	P(DAMA[HCl])	Homopolymer	82.33	23.33	Complete inhibition
P20	P(DAMA[HCl]/SO_2_ )	1:1	82.03	23.03	No inhibition
P21	P(DAMA[HCl]/AAm)	8:1	82.63	25.73	Complete inhibition
P22	P(DAMA[HCl]/MA)	N/Ds	74.7	15.70	No inhibition
Chemical structure of the repeat units: Each structure is labeled with full name of the corresponding polymers followed by their abbreviation in bracket.					
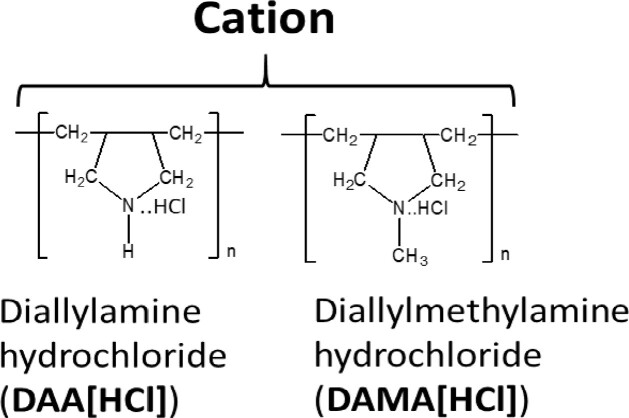		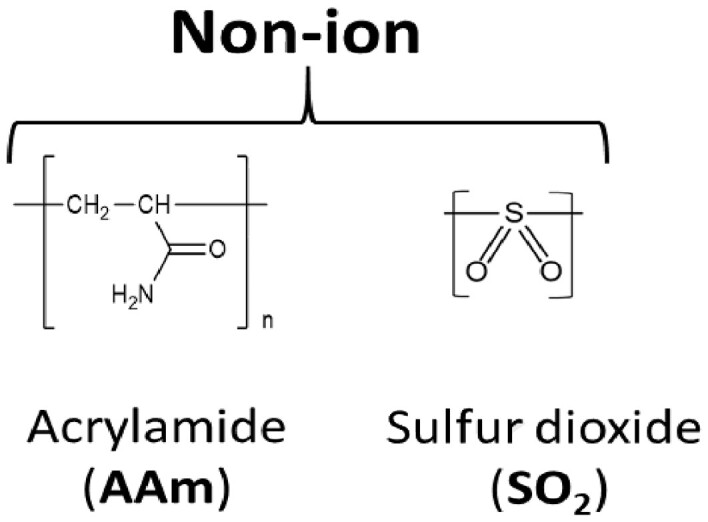		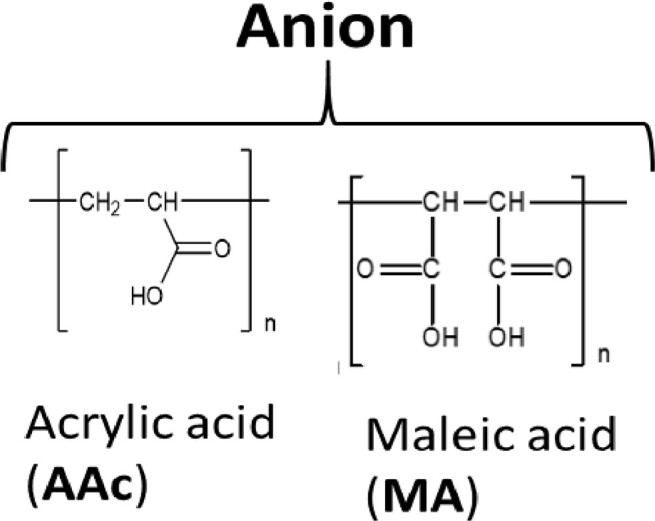	

a Ratio of monomeric precursors used in synthesis. N/Ds, not disclosed by the supplier. For the melting curve analysis, all the polymers’ concentrations were 1% (w/v). For the real-time PCR, all the polymers’ concentrations were 0.01% (w/v). *T*_m_ without polymer (reference temperature) was 58°C. *T*_m_ with 1.5 mM MgCl_2_ was 80°C.

### Real-time PCR and BNA-clamp PCR

Real-time PCR was performed on a StepOnePlus Real Time PCR system (Applied Biosystems Inc.) in a 20-µL reaction mixture containing 0.3 µM forward primer (Table 1), 0.3 µM reverse primer (Table 1), 0.01% (w/v) cationic polymer (Tables 2 and 3), 1× ROX reference dye (Takara Bio Inc.), and 1× TB Green Premix Dimer Eraser (Takara Bio Inc.), which contains TaKaRa Ex Taq^®^ HS polymerase, dNTP, MgCl_2_, TB Green dye, dimer eraser, and the template DNA, which was 2.5 ng/µL (1.65 × 10^4^ copies per reaction by calculation) for the genomic DNA and 5 × 10^5^ copies per reaction for the plasmid DNA. When plasmid DNA was used as the template, 2.5 ng/µL of salmon sperm DNA (BioDynamics Laboratory Inc.) was also included. PCR cycling conditions were as follows: 95°C (initial denaturation) for 30 s, 40 cycles of 95°C (denaturation) for 5 s, 55°C (primer annealing) for 30 s, and 72°C (extension) for 30 s. At the end of the run, melting curve analysis was performed by a subsequent single heating–cooling cycle to check if the signal was nonspecifically produced by a primer–dimer: 95°C (denaturation) for 15 s, 60°C (renaturation and dye-intercalation) for 1 min, and 95°C for 15 s.

Before the screening experiment, we initially tested varying concentrations of a few polymers that increased *T*_m_. While significant inhibition was not observed at 0.01% (w/v), they inhibited RT-PCR at concentrations between 0.1% and 1%. Therefore, we performed all the experiment of RT-PCR at 0.01% of the polymers.

BNA-clamp PCR was performed using the BNA Clamp PCR Enrichment Kit (Riken Genesis Inc.) in similar conditions as the real-time PCR above with the addition of 0.1 µM BNA clamp (Riken Genesis Inc.) and the replacement of the forward and reverse primers with those included in the kit. Polymers were added at final concentrations of 0.01–0.25% (w/v) (exact concentrations are indicated in the corresponding figure and table, which were chosen based on initial optimization with varying concentrations of these polymers). PCR cycling conditions were as follows: 95°C (initial denaturation) for 30 s, 50 cycles of 95°C (denaturation) for 20 s, 58°C (primer annealing) for 30 s, and 72°C (extension) for 45 s. Similar to the real-time PCR without BNA clamp as described above, a control melting curve analysis was also performed after the PCR reaction.

Fluorescence intensity was monitored after every cycle and threshold cycle (*C*_t_) was recorded. The data were analyzed by StepOne™ Software v2.3 and the threshold line to determine threshold cycle (*C*_t_) was automatically set at levels significantly above the background. Negative control reactions without template were included in every set of experiment and the controls gave no or significantly delayed signals indicating that no significant levels of primer dimer or non-specific products were formed during the reaction (representative data are shown in Supplementary Fig. S1).

For BNA-clamp PCR, the products were purified by a Wizard^®^ SV Gel and PCR Clean-Up System (Promega Inc.) and direct sequencing was performed (FASMAC Inc.) by Sanger sequencing using a capillary electrophoresis sequencer. Sequencing primers were included in the corresponding BNA Clamp PCR Enrichment Kit (Riken Genesis Inc.), and their sequences are not disclosed.

## Results

### Cationic polymers that increase the melting temperature of double-stranded DNA

Previous studies have indicated that the ability to stabilize dsDNA is a characteristic of copolymers, which enhances discrimination of perfectly matched and mismatched nucleotide strands [42–46]. Therefore, we first used melting curve analysis to screen a collection of cationic polymers to test their ability to increase the thermal stability of dsDNA. These polymers can be classified into the following four types: (i) poly(allylamine hydrochloride) (hereafter “P(AA[HCl])”), (ii) poly(diallylamine hydrochloride) (hereafter “P(DAA[HCl])”), (iii) poly(diallylmethylamine hydrochloride) (hereafter “P(DAMA[HCl])”), and (iv) Poly{*N*-[3-(dimethylamino)propyl](meth)acrylamide} hydrochloride (hereafter “P(DMAPMA[HCl])”) (Tables 2 and 3 and Supplementary Table S2). Many of these also contained copolymeric structures in which anionic repeat units (maleic acid or acrylic acid) and/or nonionic repeat units (sulfur dioxide or acrylamide) were incorporated between the cationic repeat units. In this system, a pair of 40-mer complementary oligonucleotides encoding part of the KRAS gene and EvaGreen, a DNA-intercalating dye, were mixed together, and different cationic polymers were added at 1% (w/v). No metal ions were added.

**Figure 1.**
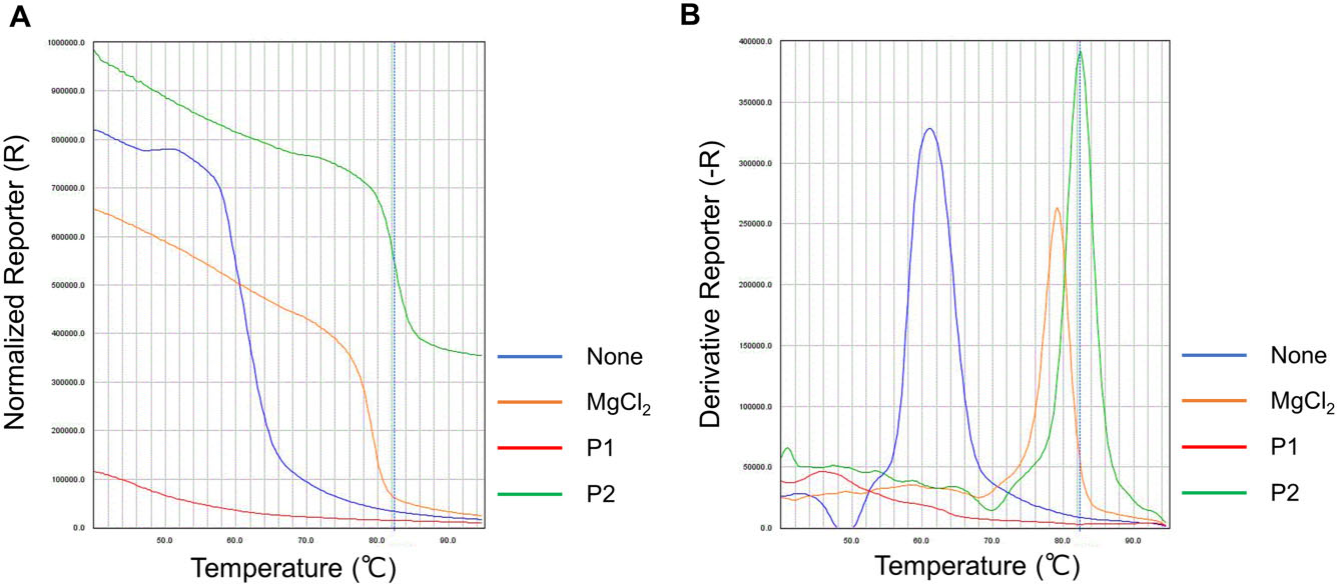
Representative melting curve profiles of the 40-mer oligonucleotides encoding a part of the KRAS gene. (**A**) Fluorescence intensity plot and (**B**) derivative plots corresponding to the addition of buffer control (i.e. no polymers), 1.5 mM MgCl_2_, 1% (w/v) P1, 1% (w/v) P2. Note that P1 showed a significantly lower fluorescence intensity below 58°C where the DNA duplex is formed even without MgCl_2_, indicating disruption of the DNA duplex.

While many of these polymers increased the *T*_m_ of dsDNA, some did not allow measurement of *T*_m_ (Tables 2 and 3 and Fig. 1). For example, in the presence of P(AA[HCl]) (P1), fluorescence intensity was lower than the control even in the temperature range in which dsDNA is formed (Table 2). The low intensity did not change significantly as the temperature increased (Fig. 1). Similar trends were also observed with P(DMAPMA[HCl]) (P3). This suggests that P(AA[HCl]) and P(DMAPMA[HCl]) inhibited the formation of dsDNA, perhaps by binding to the nucleobases of DNA.

However, when these cations formed copolymeric structures with the anionic units, they allowed for the formation of dsDNA and increased *T*_m_ (Table 2 and Fig. 1). The copolymer of P(AA[HCl]) and maleic acid increased *T*_m_ by 24°C (P2). The copolymer of P(DMAPMA[HCl]) and acrylic acid was synthesized at different ratios of cationic-to-anionic units (3:1 for P7, 1:1 for P8, and 1:3 for P9). While they all increased *T*_m_, there was a greater extent of *T*_m_ increase at higher ratios of the cationic unit. The incorporation of nonionic units (acrylamide) in P(DMAPMA[HCl]) still did not allow for the formation of dsDNA (P4, P5, and P6), while the incorporation of both nonionic and anionic units resulted in increases of *T*_m_ by 20–30°C (P10, P11, and P12), correlating with the ratio of the cationic to anionic units. As controls, we also examined the effects of polyacrylamide (nonionic unit alone, P13) and polyacrylic acid (anionic unit alone, P14), and they gave relatively small increases of *T*_m_ (by 5°C and 14°C, respectively). This suggests that, for P(AA[HCl]) and P(DMAPMA[HCl]), the coexistence of a negative charge given by the anionic units countered the inhibitory effects of cationic units and led to stabilization of dsDNA.

In contrast, P(DAA[HCl]) (P15) and P(DAMA[HCl]) (P19) both increased *T*_m_ even though they had homopolymeric structures (Table 3). These two types of cationic polymers have five-member rings consisting of four carbons (methylene) and one nitrogen, which takes the form of either a secondary or tertiary amine. For P(DAA[HCl]), the incorporation of anionic units (maleic acid for P17) as a copolymeric structure gave further increases of *T*_m_ by 3°C, but incorporation of anionic unit and nonionic unit together (acrylamide and acrylic acids for P18) gave *T*_m_ similar to the P(DAA[HCl]) homopolymer (P15). On the other hand, the incorporation of anionic units (maleic acid for P22) in P(DAMA[HCl]) resulted in an 8°C smaller increase of *T*_m_ when compared with the corresponding homopolymers of P(DAMA[HCl]). The incorporation of only nonionic units in P(DAA[HCl]) (sulfur dioxide for P16) and P(DAMA[HCl]) (sulfur dioxide for P20 and acrylamide for P21) did not have significant effects on *T*_m_.

### Compatibility of cationic polymers with PCR

**Figure 2.**
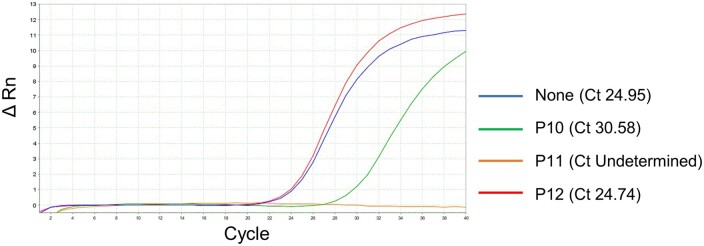
Representative real-time PCR amplification plot of KRAS gene amplification. The KRAS wild-type was amplified from genomic DNA (1.65 × 10^4^ copies per reaction) in an TB Green intercalating real-time PCR system in the presence of buffer control (i.e. no polymers) , P10 (slight inhibition), P11 (complete inhibition), and P12 (no inhibition). In this example, the final concentrations of all polymers in the reaction mixtures were 0.01% (w/v).

While the stabilizing effect of these polymers on dsDNA suggests their potential for use in rare allele detection systems, it may be important to ensure that these polymers do not interfere with PCR, which is a prerequisite for application to BNA-clamp PCR. These polymers were thus further screened based on their compatibility with PCR. As a model system, real-time PCR amplification of KRAS gene fragments was performed along with additions of the polymers. For all four types of cations, the corresponding homopolymers inhibited PCR. Nearly all the copolymers incorporating only nonionic units also inhibited PCR (Tables 2 and 3 and Fig. 2); the only exception was the copolymer of P(DAMA[HCl]) and sulfur dioxide (P20), which permitted PCR to proceed. In contrast, the copolymers incorporating anionic units (acrylic acid or maleic acid) permitted PCR. In the case of P(DMAPMA[HCl]), the extent of PCR inhibition correlated with the ratio of the cationic units in the copolymers. The copolymer gave complete or slight inhibition of PCR when the ratio of P(DMAPMA[HCl]) to acrylic acid (i.e. anionic) was 3:1 (P7) and 1:1 (P8). No inhibition was observed when this ratio was 1:3 (P9). Similarly, the copolymers incorporating both acrylamide (i.e. nonionic) and acrylic acid (i.e. anionic) (P10 and P11) showed PCR inhibition that correlated with the ratio of P(DMAPMA[HCl]) to acrylic acid (Table 2 and Fig. 2). A control polymer, polyacrylamide (nonionic unit alone, P13), gave no inhibition of PCR, but another control, polyacrylic acid (anionic unit alone, P14), gave medium levels of inhibition, which may be caused by a mechanism different from the cationic polymers. These results indicated that, with the exception of P20, the cation alone inhibits PCR, but the co-existence of an anion in the copolymers mitigates the inhibitory effect and permits PCR.

### Sequence-dependent effects of cationic copolymers on BNA-clamp PCR systems

**Figure 3.**
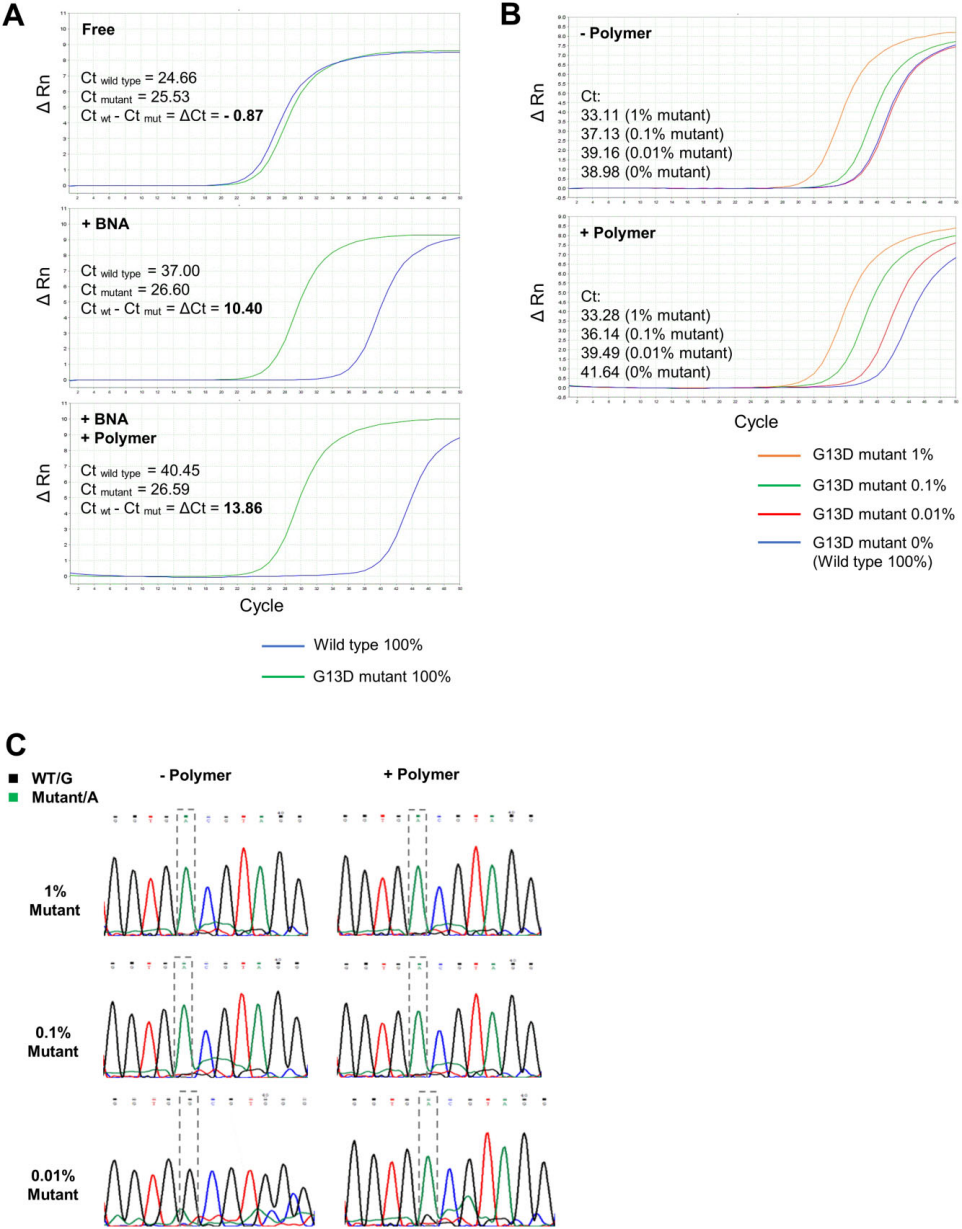
Effects of representative copolymers (P17) on wild-type-specific suppression in BNA-clamp PCR to detect KRAS G13D mutation from genomic DNA. (**A**) Real-time PCR to amplify the KRAS gene encompassing the G13 codon was performed with genomic DNA templates (1.65 × 10^4^ copies per reaction) of wild-type cells (HCC70) and mutant cells (MDA-MB-231) in the presence and absence of the BNA clamp and 0.025% (w/v) P17 polymer. (**B**) While keeping the total amount of the genomic DNA template at 1.65 × 10^4^ copies per reaction, the proportion of DNA from the mutant cells was incrementally decreased to 1%, 0.1%, 0.01%, and 0% (i.e. 100% wild-type as a reference). The BNA clamp was included in both reactions. (**C**) Direct sequencing of PCR products from the reaction shown in B at 1%, 0.1%, and 0.01% mutant-to-wild-type ratio.

In nucleic acid clamp PCR systems, increases in *T*_m_ lead to increases of specificity toward perfectly matched base pairs [32, 49]. We expected similar properties in the selected copolymers, as the polymers that increased *T*_m_ of dsDNA and permitted PCR may also increase the specificity of BNA-clamp PCR by enhancing wild-type-specific suppression. To test this, the copolymers compatible with PCR were added to a BNA-clamp PCR system designed to detect the KRAS-G13D mutation. The threshold cycle (*C*_t_) was monitored when the system was applied to genomic DNA from cultured cells carrying wild-type and mutant KRAS alleles as templates. In this experiment, we first tested the conditions in which 100% of template genomic DNA came from the cells carrying either wild-type (HCC70) or mutant (MDA-MB-231) KRAS alleles. As expected, the copolymers further suppressed wild-type KRAS allele amplification, which was indicated by increases in *C*_t_. In contrast, these copolymers had no significant effect on the mutant allele amplification (Fig. 3A). Most copolymers tested showed wild-type-specific increases in *C*_t_, although the extent of the effects varies between polymers (increases of *C*_t_ ranging by 3–10 cycles) (Table 4).

**Table 4.** Effects of the co-polymers on threshold cycle (*C*_t_) of KRAS wild-type and G13D mutant amplification in BNA-clamp PCR

Poymer number	Types of repeat units	Ratio of repeat units^a^	polymer concentration (% (w/v))^a^	100% wild-type template	100% mutant template		1% mutant template		0.1% mutant template		0.01% mutant template	
				*C*_t_	*C*_t_	Δ*C*_t_	*C*_t_	Δ*C*_t_	*C*_t_	Δ*C*_t_	*C*_t_	Δ*C*_t_
No polymer added		N/A	-	38.98	27.02	11.96	33.11	5.87	37.13	1.85	39.16	−0.18
P2	P(AA[HCl]/MA)	N/Ds	0.01	46.97	26.52	20.45	n/d	n/d	n/d	n/d	n/d	n/d
P9	P(DMAPMA[HCl]/AAc)	1:3	0.01	37.82	26.72	11.1	n/d	n/d	n/d	n/d	n/d	n/d
P12	P(DMAPMA[HCl]/AAm/AAc)	1:1:2	0.1	41.38	27.31	14.07	33.7	7.68	36.43	4.95	40.46	0.92
P17	P(DAA[HCl]/MA)	N/Ds	0.025	41.64	27.11	14.53	33.28	8.33	36.14	5.50	39.49	2.15
P18	P(DAA[HCl]/AAm/AAc)	1:1:2	0.05	41.81	28.48	13.33	35.47	6.34	39.18	2.63	40.21	1.60
P20	P(DAMA[HCl]/SO_2_ )	1:1	0.01	40.58	27.04	13.54	33.58	7.00	35.96	4.620	38.62	1.96
P22	P(DAMA[HCl]/MA)	N/Ds	0.05	46.96	26.84	20.12	33.77	13.19	35.98	10.98	37.64	9.32

a BNA-clamp was included in all the cases below. Polymer concentrations were chosen based on initial optimization of the experiments. Δ*C*_t_ = *C*_t_ 100% wild-type − *C*_t_ mutant present. N/A. not applicable. N/Ds, not disclosed by the supplier. n/d, not determined. P(AA[HCl]), poly(allylamine hydrochloride); P(DMAPMA[HCl]), poly{*N*-[3-(dimethylamino)propyl](meth)acrylamide} hydrochloride; P(DAA[HCl]), poly(diallylamine hydrochloride); P(DAMA[HCl]), poly(diallylmethylamine hydrochloride); AAm, acrylamide; AAc, acrylic acid; SO_2_, sulfur dioxide; and MA, maleic acid.

Next, we tested conditions in which mixtures of genomic DNA from the mutant and wild-type cells were used as templates to mimic clinical settings in which DNA from tumors and healthy tissues is often intermixed. While the total amount of genomic DNA template was kept constant (1.65 × 10^4^ copies per reaction), the ratio of DNA from the mutant cells was incrementally decreased, and the *C*_t_ of each reaction was compared with those cases in which 100% of the template came from the wild-type cells. Given the principle behind the BNA-clamp PCR system, in reactions lacking the copolymers, *C*_t_ increased as the mutant ratio decreased to levels similar to 100% of the wild-type (Fig. 3B and Supplementary Table S1). At a ratio of 0.01% mutant to wild-type, the *C*_t_ is indistinguishable from that of 100% wild-type.

However, when the copolymers were added to each reaction, *C*_t_ increased only when 100% of the template came from the wild-type cells. The *C*_t_ of the mutant-wild-type mixture was not significantly affected (Fig. 3B and Supplementary Table S1), rendering it possible to distinguish between 0.01% (99.99% wild-type) and 100% wild-type (Table 4). This indicated that the copolymers specifically suppressed amplification of wild-type alleles, while mutant alleles were amplified. Direct sequencing of the PCR products also showed that the addition of copolymers resulted in a smaller peak of wild-type alleles and increased the peak ratio of mutant to wild-type alleles (Fig. 3C). In the experiment above, the copy numbers of the mutant and wild-type alleles in the genomic DNA templates were calculated from the concentration of genomic DNA and average weight of DNA in the whole genome.

**Figure 4.**
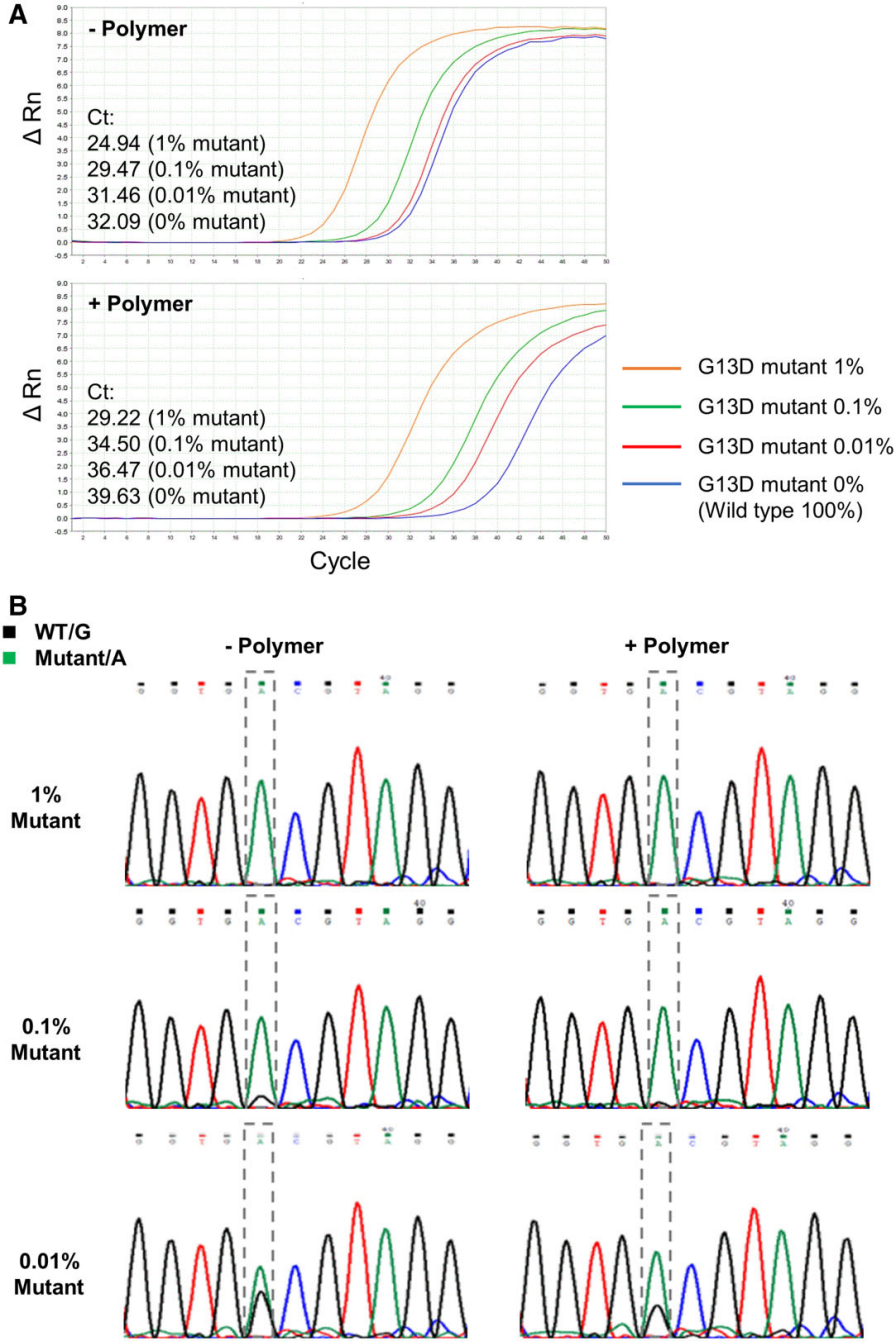
Detection limit of mutant-to-wild-type allele ratio lowered by representative copolymers (0.05% (w/v) P17) in BNA-clamp PCR when plasmid templates were used. While keeping the total concentration of the plasmid DNA template at 5 × 10^5^ copies per reaction, the ratio of mutant-to-wild-type alleles was incrementally decreased. Wild-type-specific suppression in BNA-clamp PCR was confirmed by *C*_t_ (**A**) and direct sequencing of PCR products (**B**). Similar results were obtained by the addition of P12 (Supplementary Fig. S2).

For more accurate copy numbers, we used plasmid DNA as a template and tested the effects of the copolymers in the same BNA-clamp PCR to detect the KRAS-G13D mutation. P12 and P17 were selected as representative copolymers. As was the case with the genomic DNA templates, we were not able to differentiate *C*_t_ between 0.01% mutant (i.e. 99.99% wild-type) and 100% wild-type of the KRAS plasmid templates. As expected, the addition P12 or P17 allowed distinction of *C*_t_ between 0.01% mutant and 100% wild-type (Fig. 4A, Supplementary Fig. S2A, and Supplementary Table S1). However, in case of the plasmid DNA, *C*_t_ also increased with the templates containing mutant alleles (1%, 0.1%, and 0.01% alleles), although the degree of the increase was less than that of the 100% wild-type template. This indicated that, in case of the plasmid templates, these copolymers suppressed PCR to a certain extent even when the template DNA contained the mutant allele, but PCR of the wild-type allele was more preferentially suppressed. Direct sequencing of the PCR product also showed that amplification of the wild-type allele was more preferentially suppressed when the copolymers were added (Fig. 4B and Supplementary Fig. S2B).

**Figure 5.**
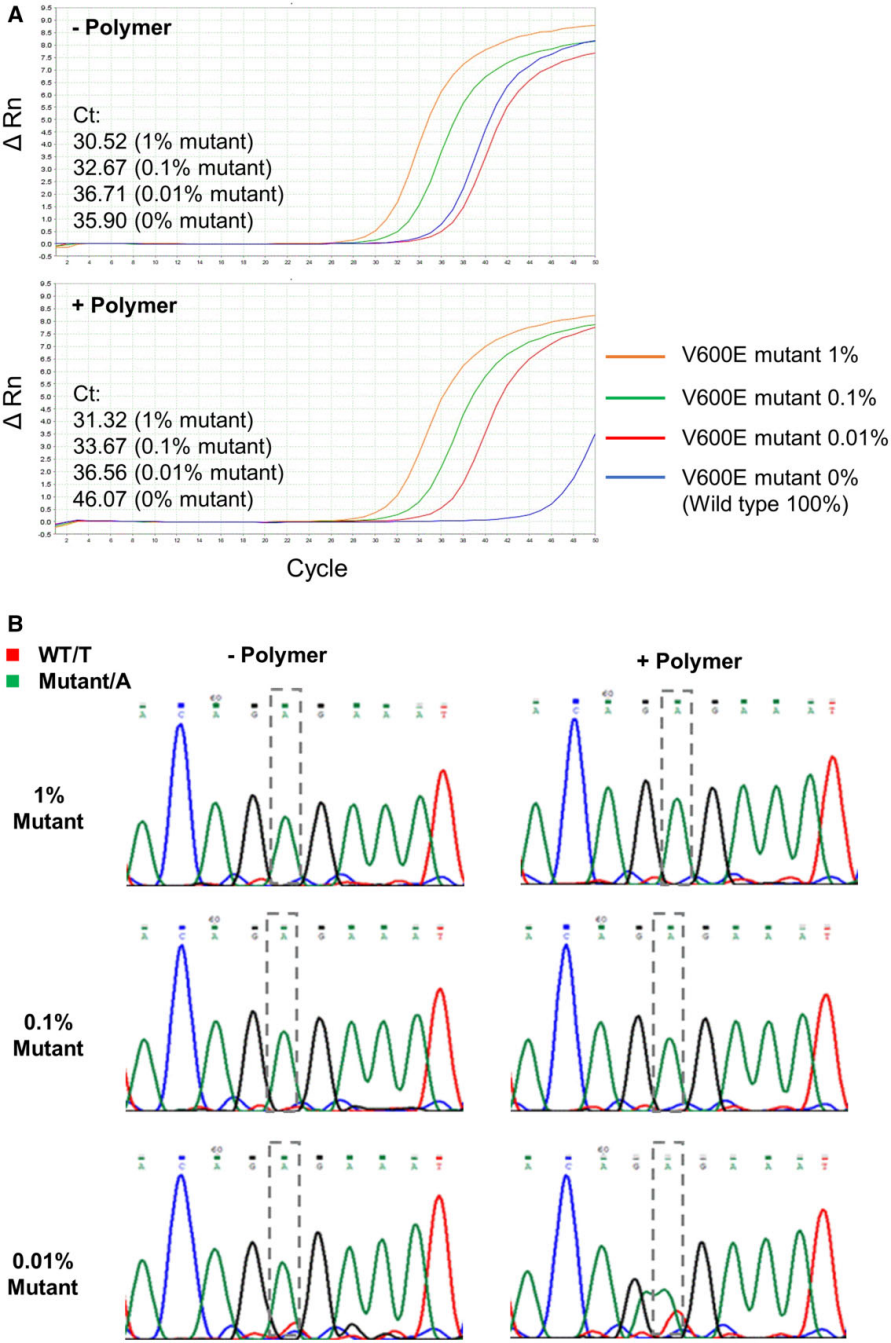
Effects of representative copolymers (0.05% (w/v) P17) on wild-type-specific suppression in BNA-clamp PCR to detect BRAF alleles from genomic DNA. While keeping the total amount of genomic DNA template at 1.65 × 10^4^ copies per reaction, the proportion of DNA from the mutant cells (DU4475) over the wild-type cells (HCC70) was incrementally decreased to 1%, 0.1%, 0.01%, and 0% (i.e. 100% wild type as a reference). Wild-type-specific suppression in BNA-clamp PCR was confirmed by *C*_t_ (**A**) and direct sequencing of PCR products (**B**). Similar results were obtained by the addition of P12 (Supplementary Fig. S3).

To test if the copolymers show wild-type-specific suppression of PCR for other genes, the selected copolymers were added to a BNA PCR clamping system designed to detect the BRAF V600E mutant. Genomic DNA from cultured cells carrying wild-type (HCC70) and mutant (DU4475) BRAF alleles was used as the template. Similar to the KRAS system, the addition of copolymers further increased the *C*_t_ of the BRAF wild-type by 3–10 cycles, but no significant changes were found in the *C*_t_ of the mutant alleles (Fig. 5A, Supplementary Fig. 3A, and Supplementary Table S1). These copolymers also did not affect *C*_t_ significantly when mixtures of genomic DNA from the mutant and wild-type cells were used as templates. When the copolymers were not added, the *C*_t_ of 0.01% mutant and 100% wild-type were almost indistinguishable, but the addition of these copolymers allowed us to distinguish between 0.01% mutant and 100% wild-type (Fig. 5A, Supplementary Fig. S3A, and Supplementary Table S1). On the other hand, direct sequencing of the PCR product showed amplification of the mutant allele at 1%, 0.1%, and 0.01% mutant concentrations even in the absence of the copolymers (Fig. 5B and Supplementary Fig. S3B). A previous study using BNA-clamp PCR also reported similar observation with a few samples in which mutations were detected by the direct sequencing but not by the amplification plot [39]. Nevertheless, these data suggested that wild-type-specific suppression by these copolymers in BNA PCR clamping is not limited to the KRAS gene, but is generally applicable to other genes.

### Sequence-dependent effects of cationic polymers on the *T*_m_ of DNA–DNA and BNA–DNA complementary strands

As the selected copolymers promoted discrimination between mutant and wild-type alleles in BNA-clamp PCR, we sought a mechanistic model that can explain the polymers’ effects. For nucleic acid clamps, the structural stability of synthetic nucleotides preferentially increases the *T*_m_ of perfectly matched complementary sequences (wild-type) over those containing a mismatch (mutant), resulting in an increased difference (i.e. Δ*T*_m_ = *T*_m wild-type_−*T*_m mutant_) [32, 49]. By following these previous reports, we tested whether the representative polymers (P12 and P17) have similar properties.

**Figure 6.**
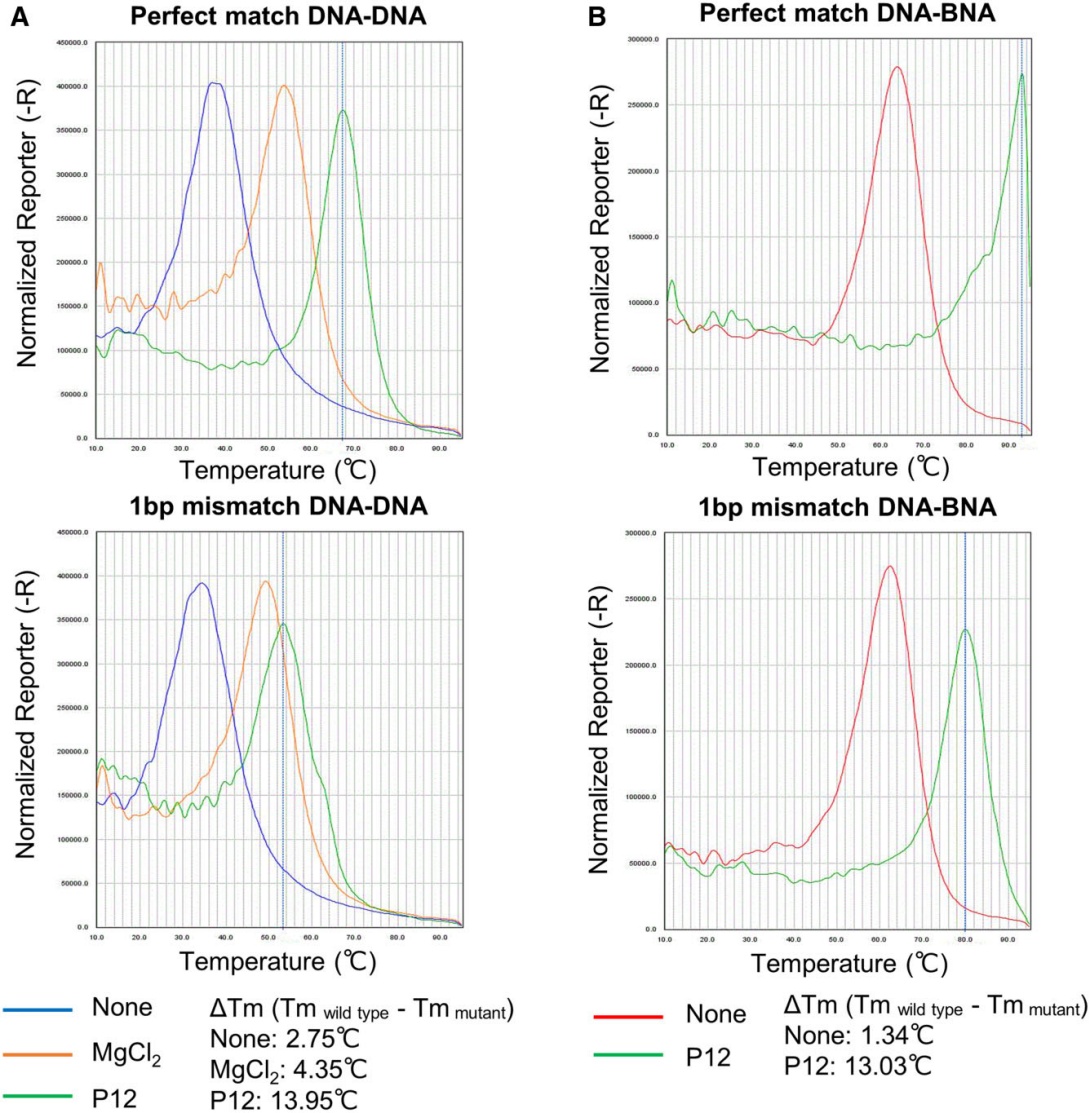
Melting curve analysis of 11-mer complementary strands encompassing the KRAS G13D mutation site. (**A**) Profiles from DNA–DNA strands with a perfect match (wild-type) and a single nucleotide mismatch (mutant) with the addition of P12. (**B**) Profile from the DNA–BNA strands equivalent to (A).

First, a pair of 11-mer DNAs encoding the sense strand of the KRAS wild-type or KRAS G13D mutant encompassing position 13, and its complementary antisense DNA from the KRAS wild-type strand, were tested in pairs (i.e. sense–antisense DNA pairs of wild-type–wild-type or G13D mutant–wild-type). In the absence or presence of the copolymers, melting curve analyses were performed to obtain the *T*_m_ of each condition. Although the *T*_m_ of both wild-type–wild-type and G13D mutant–wild-type pairs increased by the addition of polymer, a significantly higher degree of *T*_m_ increases was found for the wild-type–wild-type pair (Fig. 6A and Table 5). As a result, their Δ*T*_m_ (*T*_m wild-type_−*T*_m mutant_) increased (Table 5).

**Table 5.** Summary of *T*_m_ in the melting curve analysis of the DNA–DNA and DNA–BNA 11 mer complementary strands (summary of Fig. 6)

Poymer number	Types of repeat units	Ratio of repeat units^a^	DNAwt–DNAwt	DNAmut–DNAwt	Δ*T*_m_ (°C) (DNA-DNA)	DNAwt–BNAwt	DNAmut–BNAwt	Δ*T*_m_ (°C) (DNA–BNA)
			*T*_m_ (°C)	*T*_m_ (°C)		*T*_m_ (°C)	*T*_m_ (°C)	
No polymer added		N/A	37.00	34.25	2.75	63.74	62.4	1.34
P12	P(DMAPMA[HCl]/AAm/AAc)	1:1:2	67.30	53.35	13.95	92.95	79.92	13.03
P17	P(DAA[HCl]/MA)	N/Ds	69.85	56.05	13.80	92.95	81.10	11.85

Antisense strands were wild-type DNA or BNA in all the cases. See Table 1 for the sequences. Δ*T*_m_ = *T*_m wild type_−*T*_m mutant_. N/Ds, not disclosed by the supplier. P(DMAPMA[HCl]), poly{*N*-[3-(dimethylamino)propyl](meth)acrylamide} hydrochloride; P(DAA[HCl]), poly(diallylamine hydrochloride); AAm, acrylamide; AAc, acrylic acid; MA, maleic acid.

Second, a similar experiment was performed by replacing the antisense strand with an oligonucleotide mainly consisting of BNA (i.e. wild-type or G13D sense strand DNA and complementary wild-type strand, of which 8 nucleotides were BNA and 3 nucleotides were DNA) (Table 1). Similar to the case of the DNA–DNA pairs, the addition of the copolymers preferentially increased the *T*_m_ of the wild-type DNA–wild-type BNA pairs when compared with the G13D mutant DNA–wild-type BNA pairs (Fig. 6B and Table 5), resulting in increases of Δ*T*_m_ (*T*_m wild-type_−*T*_m mutant_) (Table 5).

These results corroborate the explanation that the polymers’ mode of action in BNA-clamp PCR is similar to the mechanism of PCR clamping to discriminate wild-type and mutant alleles by increasing the stability of hybridization.

## Discussion

In this study, we identified cationic polymers that can increase the *T*_m_ of complementary nucleotide strands and enhance the detection of rare alleles in BNA-clamp PCR. The cationic repeat units of these polymers consisted of four types of amine groups, and the majority of them had copolymeric structures incorporating anionic and/or nonionic repeat units. While a total of 17 types of polymers increased the *T*_m_ of the complementary strands, only 8 of these polymers permitted PCR. With one exception (P20), most of these 8 polymers were copolymers of cationic and anionic units. The majority of these copolymers also showed enhancement of wild-type-specific suppression in BNA-clamp PCR.

Previous studies have shown that cationic polymers such as poly-lysine [42, 50], poly-arginine [51], and polyamine [52–54] bind to DNA and increases *T*_m_. For poly-lysine, its binding to DNA causes the formation of insoluble polymer–DNA complexes that prevent the denaturation of dsDNA into ssDNA [43, 50]. However, when a large fraction (>80%) of ε-amino groups of poly-lysine are grafted with dextran, the resulting copolymer forms a soluble complex with DNA and allows for the denaturation and renaturation of target dsDNA [43]. Similar to these studies, our polymers consisting of only P(AA[HCl]) and P(DMAPMA[HCl]) disrupted dsDNA formation, rendering it impossible to measure *T*_m_. A possible explanation is that cation of these copolymers can potentially interact with the nucleotide bases and disrupt the formation of base pair. Regarding P(AA[HCl]), its primary amine can also form hydrogen bonding if positioned properly. However, when anionic units (maleic acid or acrylic acid) were incorporated in P(AA[HCl]) and P(DMAPMA[HCl]), the resulting copolymers allowed for the formation of dsDNA, resulting in increases of *T*_m_. Incorporation of anionic units might have neutralized some amines and prevent the disruption of ddDNA formation. Increases of *T*_m_ were also observed with homopolymers of P(DAA[HCl]) and P(DAMA[HCl]). When compared with P(AA[HCl]) and P(DAMAPMA[HCl]), cationic units of these polymers are structurally less flexible. They may be more constrained and not allowed to reach the nucleotide bases. Nevertheless, *T*_m_ was still affected by the incorporation of nonionic or anionic units. The latter observation is similar to previous studies with copolymers of poly-lysine grafted with dextran or guanidine [43, 55]. In these studies, the fraction or size of grafted moieties intercalating the lysine repeat units significantly affects *T*_m_. Regarding the molecular design of copolymers that modulate *T*_m_, one major addition of our study to previous studies [42, 55] is the incorporation of anionic units into the cationic polymers, which significantly affected the formation of dsDNA and increases in *T*_m_.

To apply the *T*_m_-modulating polymers to mutant detection, we sought PCR-based methods. While some studies have applied cationic copolymers to mutant detection by methods without PCR, such as a FRET-based ssDNA exchange system [45, 56] and multicomponent nucleic acid enzymes [57], we thought that compatibility with PCR can facilitate broad applications. All the copolymers that permitted PCR increased the *T*_m_ by 15–23°C. However, the polymers that increased *T*_m_ by more than 25°C interfered with PCR, suggesting that too much of an increase in *T*_m_ leads to interference, perhaps by prohibiting denaturation of dsDNA. Similar to our observation, a previous study using various copolymers of poly-lysine showed an inverse relationship between the degree of *T*_m_ increase and strand exchange reactions of dsDNA and ssDNA [55]. While the stability of dsDNA promotes certain reactions involving DNA, moderate levels of stability seem to be appropriate for PCR to take place. However, the degree of *T*_m_ increase alone may not be a sufficient criterion for compatibility with PCR. Homopolymers of P(DAA[HCl]) and P(DAMA[HCl]) (i.e. P15 and P19) and a copolymers of P(DAA[HCl]) and non-ionic units (i.e. P16) increased *T*_m_ by 19–23°C, but they still inhibited PCR. When anionic units were incorporated in P(DAA[HCl]) and P(DAMA[HCl]), the resulting copolymers permitted PCR. Although incorporation of anionic unit is not always required for compatibility with PCR (e.g. P20), it might neutralize inhibitory effects of the cationic units. For example, it is possible that cationic units alone block DNA elongation or inhibit DNA polymerase even when they allow formation of dsDNA.

**Figure 7.**
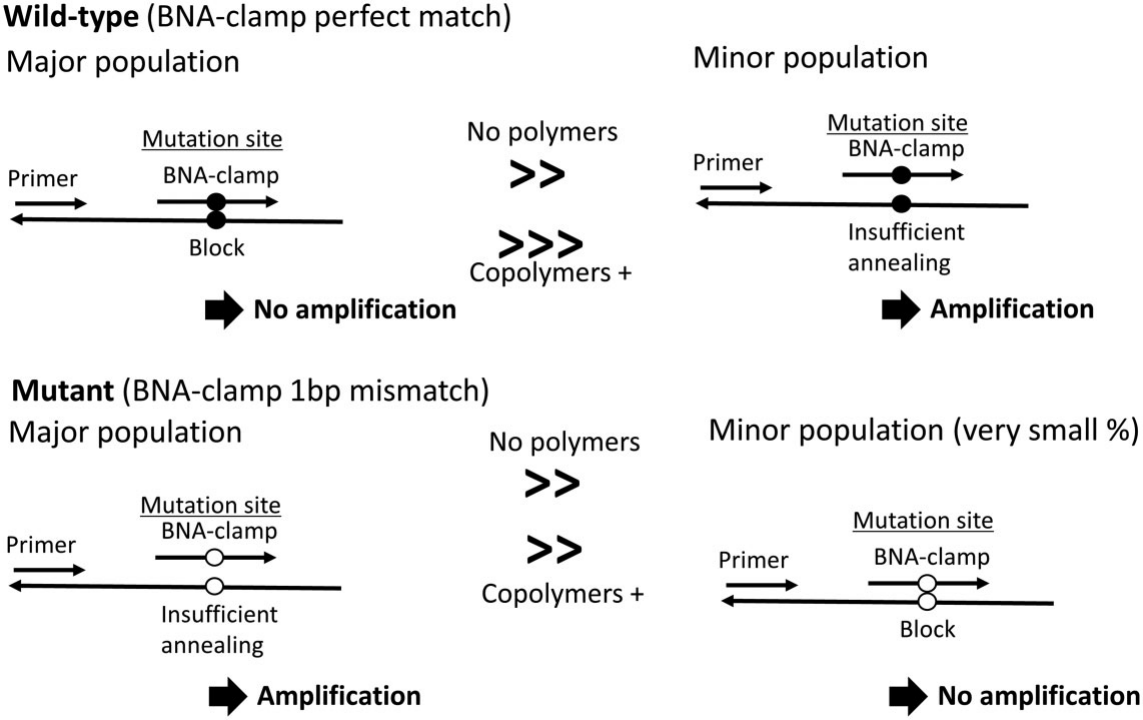
A model for enhancement of preferential suppression of wild-type alleles in BNA-clamp PCR by addition of the copolymers. Although BNA-clamp PCR preferentially blocks amplification of wild-type allele, minor the population is still unblocked and amplified. Addition of the copolymers further increases relative population of wild-type allele blocked by BNA-clamp PCR. Nearly all of mutant allele is unblocked by BNA-clamp PCR and the copolymers does not affect mutant significantly.

Although our study did not elucidate the mechanisms of the enhanced discrimination of mutant and wild-type alleles in BNA-clamp PCR, some data gave clues for further investigation. The melting curve analyses with 11-mer DNA–DNA and BNA–DNA complementary strands (Fig. 6) indicated that the selected copolymers preferentially stabilized perfectly matched strands when compared with the mismatched strand by increasing *T*_m_ to a higher degree. Based on this observation, we propose a model (Fig. 7) for how the preferential stabilization of perfectly matched strands leads to enhancement of mutant detection in BNA-clamp PCR. Even though BNA-clamp is designed to block amplification of the wild-type allele, it may still leave a minor population of wild-type allele unblocked and allow their amplification. The copolymers further enhance blocking of wild-type alleles and leave smaller population of the unblocked wild-type allele. Although copolymers can also stabilize hybridization of BNA-clamp to the mutant allele, its effects on the mutant allele are not as significant, leaving the mutant allele unblocked. This model is not fully supported by the melting curve analysis with the 11-mer oligonucleotides as *T*_m_ of 11-mer BNA-mutant DNA strands was higher (79–80°C) than annealing and elongation temperature (55°C and 72°C). However, the balance between the blocked and unblocked population can also be affected by differences in concentration of the copolymers, buffer conditions, and existence of competing wild-type alleles, and we believe that this model roughly represents the copolymers’ mode of action on BNA-clamp PCR.

The mode of action shown in this model resembles the mechanisms of mismatch/perfect match discrimination enhanced by BNA or LNA (early generation BNA with an unmodified 2′ oxygene and the 4′ carbon bridge is often called LNA). A systematic comparison of *T*_m_ for a perfectly matched and 1-bp mismatched DNA–LNA-containing strand (triplet LNA flanked by DNA) and DNA–DNA double strands [58] showed that increases in *T*_m_ by inclusion of LNA is driven by a gain of enthalpy, possibly due to the enhancement of base-stacking and hydrogen bonding. Such enhancement is likely to be lost by mismatch, leading to significant destabilization and decreases in *T*_m_. Based on the similarity of LNA and RNA in the loss of free energy by specific types of mismatches, the authors hypothesized that LNA’s mismatch discrimination originated from B-form to A-form transition of the double-strand conformation and that this mode of discrimination is not unique to LNA [58]. Interestingly, Yamaguchi *et al.* [59] reported that the binding of dextran-grafted polyarylamine to some 10-bp G–C rich dsDNA converts their structures from B-form to A-form, most likely by dehydrating DNA. Although still speculative, we hypothesize that our polymers induce B-form to A-form transition of the target DNA, strengthen base-pair hydrogen bonding and base-stacking, and thereby enhance mismatch/perfect match discrimination. For BNA–DNA double strands in our system, BNA may already provide such an enhancement. However, our polymers may still act on the DNA strand complementary to BNA, resulting in an additive enhancement of discrimination. Secondary structural and thermodynamics analyses of DNA and BNA upon the addition of our polymers might give further clues to understand the mechanism.

So far, we have tested the effects of the copolymers on BNA-clamp PCR for KRAS and BRAF mutations with genomic DNA and plasmid templates. Although our results suggest the enhancement of wild-type-specific suppression in BNA-clamp PCR in general, further evaluations of these polymers are needed to learn how versatile they are. For example, a BNA-clamp PCR system has been developed to detect single-nucleotide substitutions of other oncogenes such as EGFR [60] and JAK2 [61]. Instead of a building block of a clamp to block amplification of an undesired allele, BNA (often called LNA) is also incorporated in FRET-based detection probes that have higher specificity than similar probes consisting of only DNA [62, 63] and our polymers will likely further enhance the specificity of such probes. In order for such probes to give off signals, our copolymers need to allow DNA polymerase to cleave the probes by its exonuclease activities. Our pilot study with P17 and P12 showed that these polymers are, at least, compatible to a TaqMan real-time PCR system (Supplementary Fig. S4). In addition, in clinical settings, the template DNAs for mutant detection by BNA clamp PCR are extracted from FFPE tissues [39, 41], tissue biopsies [40], or blood (i.e. cell-free DNA) [40, 61]. Further evaluation of our copolymers with different types of PCR systems utilizing BNA and different sources of template DNA will reveal how applicable they are in clinical settings.

While BNA-clamp PCR is mostly used for research purposes, its clinical adaptation for *in vitro* diagnostics would give additional options for cancer diagnosis with high usability [39] and versatility [22, 40, 61]. BNA-clamp PCR combined with downstream detection systems has sensitivities of a mutant-to-wild-type ratio of 0.1–5% [39, 40, 60], which is comparable to other rare allele enrichment systems [3, 4, 14, 15, 29]. In our experiments, the addition of copolymers specifically increased the *C*_t_ of the wild-type alleles in BNA-clamp PCR by 3–10 cycles, which is equivalent to an increase of sensitivity by 10- to 100-fold. This would potentially position BNA-clamp PCR as one of the most sensitive methods of choice. In practice, our copolymers can potentially be used as an additive to existing BNA-clamp PCR without changing their compositions.

Our observation of preferential increases in *T*_m_ for perfectly matched complementary strands in the melting curve analysis suggested potential applications of these copolymers to other systems for rare allele detection. For example, melting curve analysis itself has been used as a detection method with samples in which target fragments of mutant and wild-type coexist [13, 64]. The addition of our copolymers may readily increase the sensitivity for mutants in these systems. AS PCR has been developed for clinical use [14, 15, 65], but this method is prone to false-positive results due to nonspecific annealing of the primers [14, 15, 66]. It is possible that the addition of our copolymers can preferentially stabilize the annealing of primers with fewer mismatches and decrease the chances of false-positive results. In addition to BNA-clamp PCR, other types of techniques have been developed to preferentially suppress amplification of wild-type allele [23], including oligoribonucleotide interference-PCR (ORNi-PCR) [25, 67] and blocking oligonucleotide PCR [68]. As their mode of action and chemical structures of the wild-type blocking strands (e.g. RNA and dideoxynucleotide) are similar to BNA-clamp, inclusion of our copolymers in these systems might also enhance detection of the rare alleles. Despite various potential applications of our copolymers, however, our pilot study with a PNA-clamp PCR kit for the detection of the KRAS mutation (PANAGENE Inc.) showed that the addition of our copolymers suppressed PCR for both wild-type and mutant alleles to some degree in the presence of the PNA clamp (Supplementary Fig. S5). This indicates that our copolymers can interact with molecules other than DNA and BNA, but they can be inhibitory. It is worth pursing further testing of our copolymers with other mutant detection systems, perhaps at various concentrations, to see whether or not they can give preferential effects.

## Accession numbers

No public repository accession numbers are accompanied with this manuscript.

## Supplementary data

Supplementary data are available at *Biology Methods and Protocols* online.

## Data availability

The data underlying this article are available in the article and in its online supplementary material.

## Funding

This study was entirely supported by internal fundings of Nitto Boseki Co., Ltd. and Nittobo Medical Co., Ltd. spcifically allocated for research and development activities of Nitto Boseki Co., Ltd. and Nittobo Medical Co., Ltd. in the fiscal years 2019, 2020 and 2021.

## Conflict of interest

The authors of this article have filed patent applications related to the copolymers that increase *T*_m_ and enhance detection of the rare alleles by BNA-clamp PCR with following details; (i) Agents that increase melting temperature (*T*_m_) of nucleic acid, Japan Patent Office application No. 2021-201160; (ii) Agents that increase melting temperature of double-stranded nucleic acids and their applications, Japan Patent Office application No. 2021-201162; (iii) Methods to detect mutation in the sequence of target nucleic acid strands and kit to apply the said methods, Japan Patent Office application No. 2021-201175; and (iv) Methods to detect mutation in the sequence of target nucleic acid strands and kit to apply the said methods, Japan Patent Office application No. 2021-201177.

## Supplementary Material

bpac009_Supplementary_Data
