# Supplementary material for: Cationic copolymers that enhance wild-type-specific suppression in BNA-clamp PCR and preferentially increase the Tm of fully matched complementary DNA and BNA strands
Source: Biol Methods Protoc. 2022 Mar 30;7(1):bpac009. doi: 10.1093/biomethods/bpac009 (PMC9154250; doi:10.1093/biomethods/bpac009)
Supplement: bpac009_Supplementary_Data [file bpac009_supplementary_data.pdf]

SUPPLEMENTARY DATA

Dataset below may help readers to further comprehend the main body of this manuscript.

Supplementary Table 1. Summary of three repeats of BNA-clamp PCR

Experiments shown in Figure 3, Figure 4 and Figure 5 were repeated three times (i.e. n=3).

Average Ct ±Standard deviation of the three independent experiments are listed.

(Including data from the experiment shown in Figure 3, Figure 4 and Figure 5)

BNA-clamp PCR to detect G13D KRAS mutant from the genomic DNA

(Corresponding to Figure 3)

Poymer number	Types of repeat units	Ratio of repeat units*	1% mutant template	0.1% mutant template	0.01% mutant template	100% wild type template
			Ct	Ct	Ct	Ct
No polymer added		N/A	32.8 ±0.24	36.64 ±0.47	42.07 ±3.33	39.85 ±0.94
P12	P(DMAPMA[HCl]/AAm/AAc)	1:1:2	32.79 ±0.82	35.79 ±0.56	38.02 ±1.81	44.47 ±2.77

BNA-clamp PCR to detect G13D KRAS mutant from the plasmid DNA

(Corresponding to Figure 4 & Supplementary Figure 2)

Poymer number	Types of repeat units	Ratio of repeat units*	1% mutant template	0.1% mutant template	0.01% mutant template	100% wild type template
			Ct	Ct	Ct	Ct
No polymer added		N/A	27.75 ±2.56	30.77 ±0.93	31.75 ±0.21	32.32 ±0.17
P12	P(DMAPMA[HCl]/AAm/AAc)	1:1:2	30.46 ±1.00	34.57 ±0.29	36.68 ±0.37	38.53 ±0.88
P17	P(DAA[HCl]/MA)	N/Ds	28.94 ±1.26	32.58 ±1.02	34.24 ±0.77	35.32 ±0.77

BNA-clamp PCR to detect V600E BRAF mutant from the genomic DNA

(Corresponding to Figure 5 and Supplementary Figure 3)

Poymer number	Types of repeat units	Ratio of repeat units*	1% mutant template	0.1% mutant template	0.01% mutant template	100% wild type template
			Ct	Ct	Ct	Ct
No polymer added		N/A	31.24 ±0.52	34.22 ±1.40	38.88 ±2.44	38.01 ±1.56
P12	P(DMAPMA[HCl]/AAm/AAc)	1:1:2	31.77 ±0.33	35.3 ±1.16	39.52 ±3.50	45.01 ±1.30
P17	P(DAA[HCl]/MA)	N/Ds	32.43 ±0.85	36.16 ±1.17	39.68 ±1.44	44.71 ±3.00

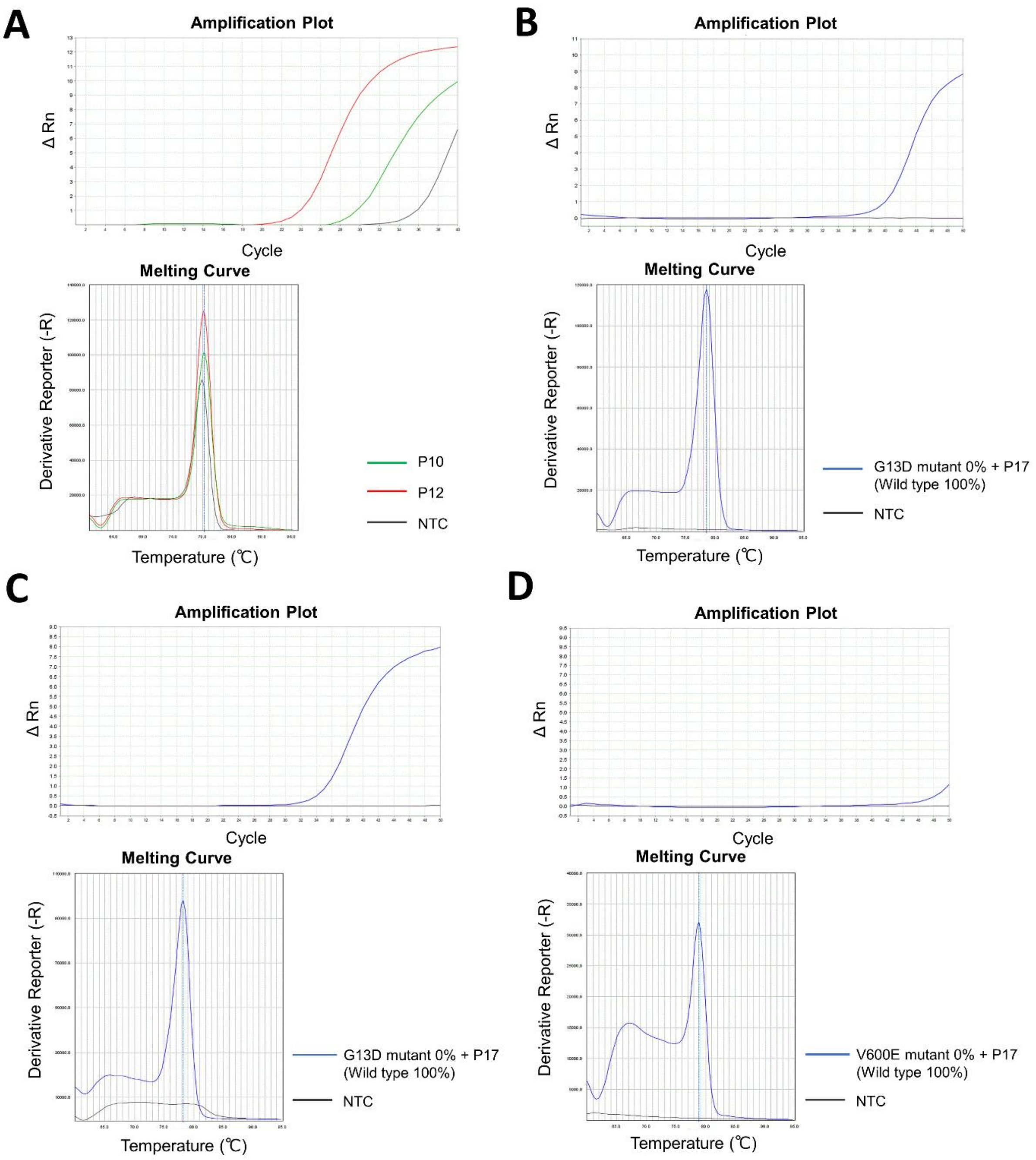

Supplementary Figure 1. Data from the non-template negative controls (NTC). In all the experiments of RT-PCR and BNA-clamp PCR, negative controls without template were included to ensure amplification of the specific target. For comparison data from 100% wild type template with one or two representative polymers are superimposed. No or significantly delayed amplification (upper panel) was observed in NTC in; A) RT-PCR screening shown in Figure 2, B) BNA-clamp PCR to detect KRAS G13D mutation from the genomic DNA template shown in Figure 3A, C) BNA-clamp PCR to detect KRAS G13D mutation from the plasmid template shown in Figure 4A and D) BNA-clamp PCR to detect BRAF V600E mutation from the genomic DNA template shown in Figure 5A. Post PCR melting curve analysis corresponding to these experiments (lower panel) also indicated no or significantly low levels of product formation of in the non-template control and formation of single *Tm* product in the reaction with the template.

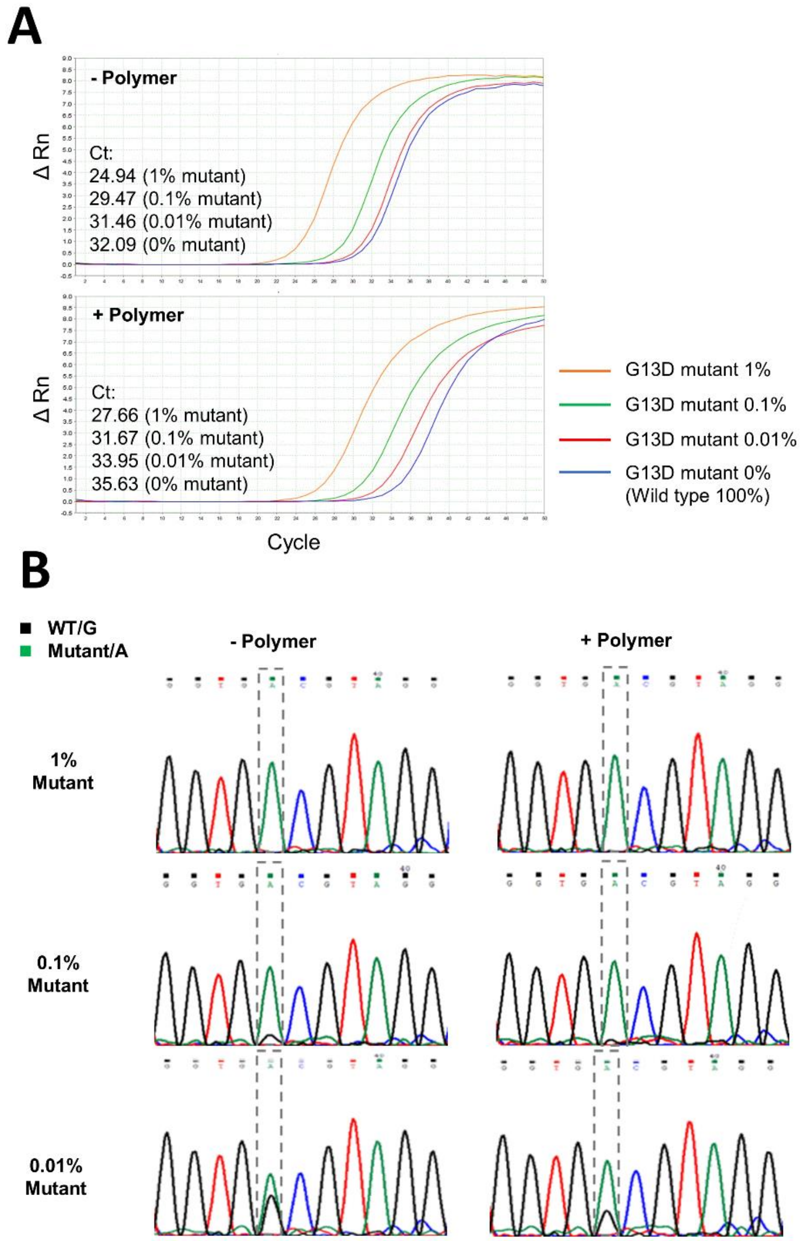

Supplementary Figure 2. Detection limit of mutant to wild type allele lowered by representative copolymers (0.25% (w/v) P12) in BNA-clamp PCR when plasmid templates were used. The same experiment as in Figure 4 was performed by addition of P12 copolymer; A) Amplification plot and B) Direct sequencing of PCR products in A.

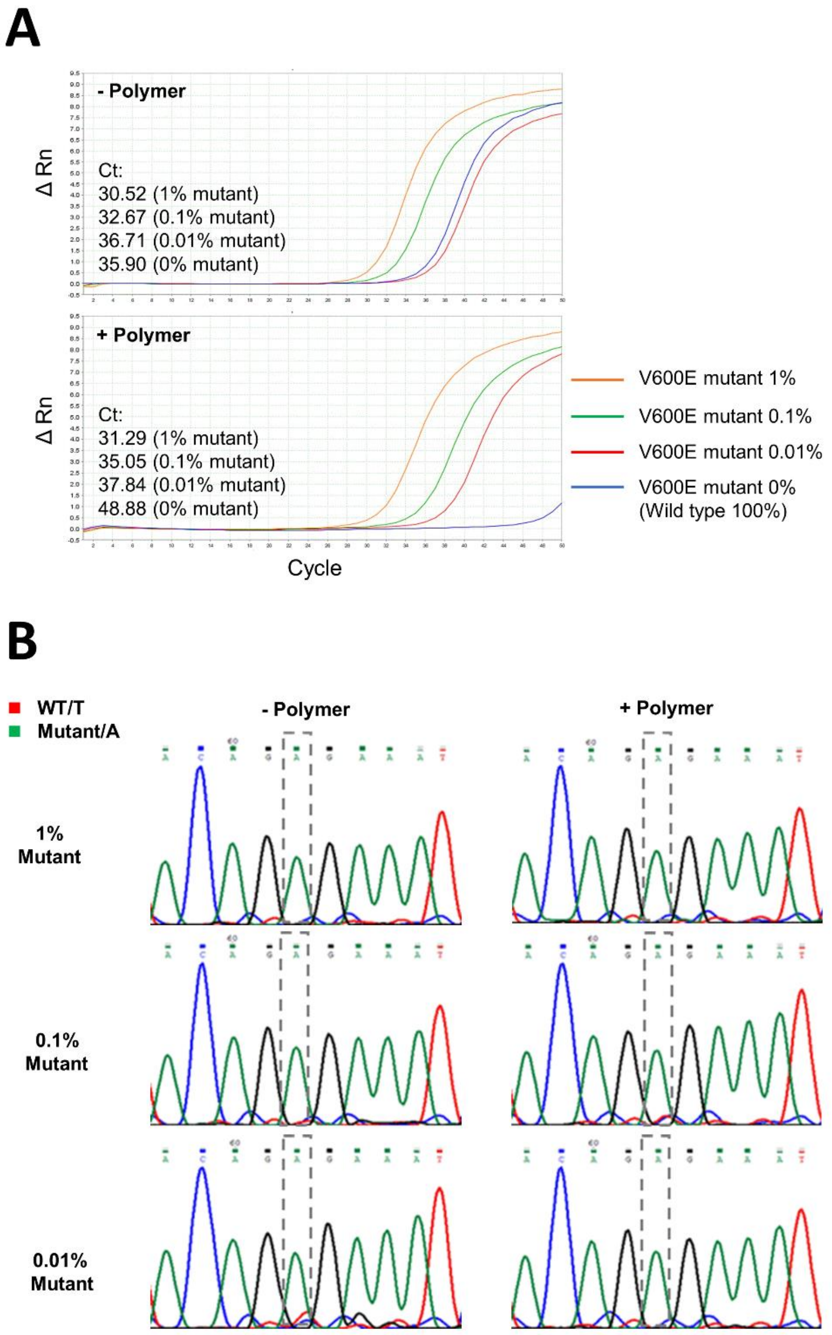

Supplementary Figure 3. Effects of representative copolymers (0.1% (w/v) P12) on wild type specific suppression in BNA-clamp PCR to detect BRAF alleles from genomic DNA. The same experiment as in Figure 5 was performed by addition of P12 copolymer; A) Amplification plot and B) Direct sequencing of PCR products in A.

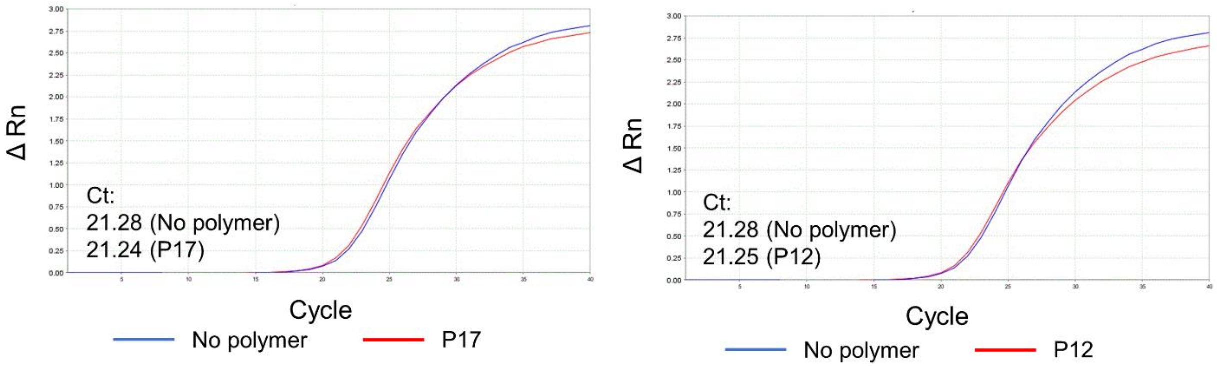

Supplementary Figure 4. Compatibility of copolymers with TaqMan real time PCR. Two representative polymers, P17 & P12, were added to a real time PCR with TaqMan probe detection system at 0.01% and 0.1% respectively. (Reference reaction of TaqMan Mutation Detection Assay (Thermo Fisher Scientific) was performed by following manufacturer`s instruction. At these concentrations, these two copolymers do not inhibit BNA-clamp PCR to detect KRAS G13D mutant from genomic DNA.) 50ng (1.65 x 10^4 copies) per reaction Genomic DNA from HCC70 cells were used for each reaction.

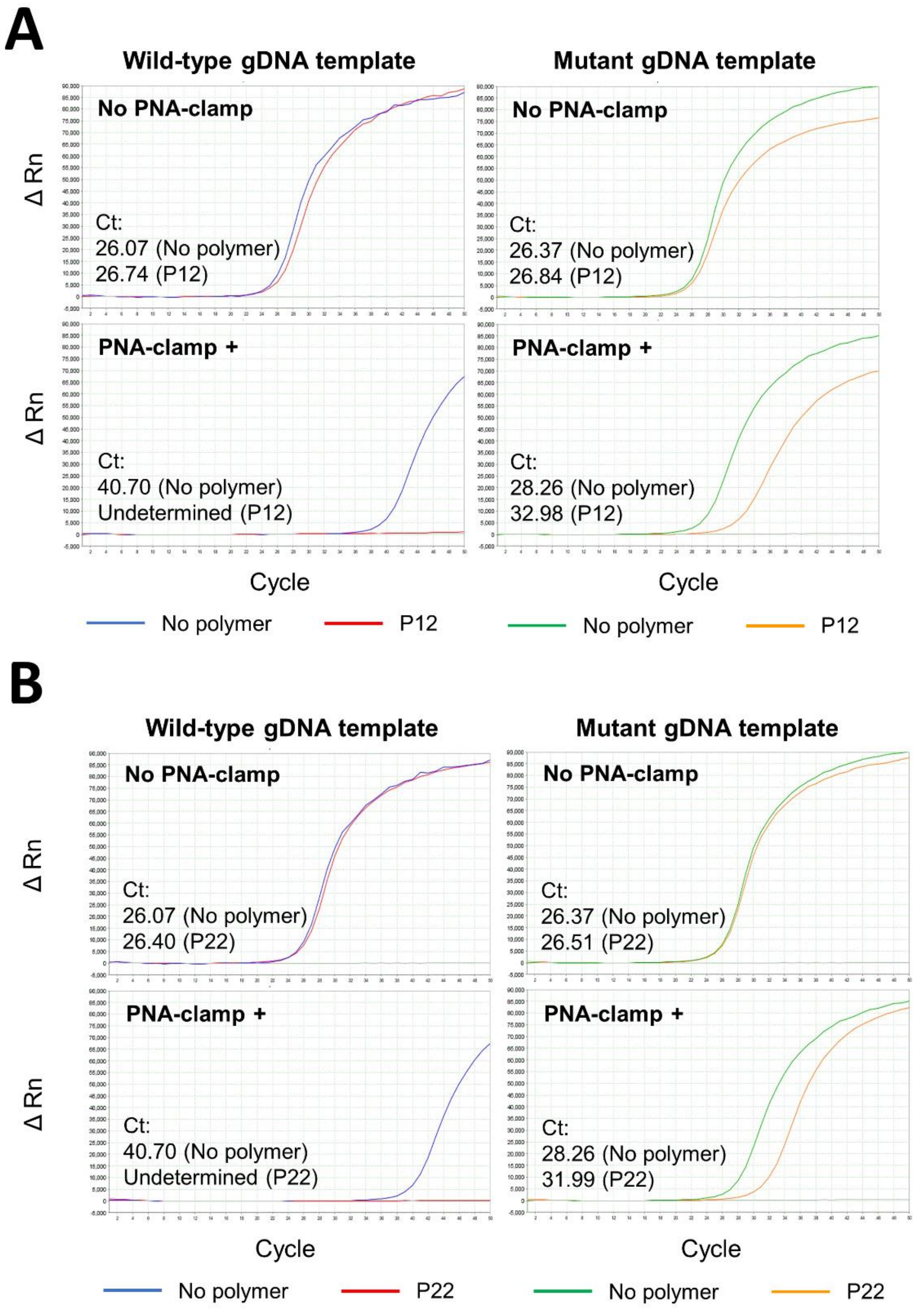

Supplementary Figure 5. Effects of a representative copolymer (P12) on PNA-clamp PCR. KRAS fragment encompassing G13 was amplified in the presence and absence of PNA targeting G13 and 0.2% (w/v) P12 (A) or 0.1% (w/v) P22 (B). PANA Clamp KRAS Mutation Detection Kit (PANAGENE) was used by following manufacturer`s instruction. (At these concentrations, these two copolymers do not inhibit BNA-clamp PCR to detect KRAS G13D mutant from genomic DNA.) 10ng (3.3 x 10^3 copies) per reaction of genomic DNA from HCC70 and MDA-MB-231 cell lines were used as wild-type and mutant template, respectively.

Supplementary materials for synthesis of the polymers

The polymers described herein were specifically synthesized for this study by the methods below. As this piece of information may take up space and can be redundant, they are included it in the supplementary data section. Nevertheless, we still consider it to be helpful for better comprehension and future reproducibility.

Materials for synthesis of polymers

N-[3-(dimethylamino)propyl](meth)acrylamide, diallylamine hydrochloride, ammonium persulfate, acrylamide, and acrylic acid were purchased from Tokyo Chemical Industry Co. Ltd. (Tokyo, Japan). Diallylmethylamine was purchased from Wako Pure Chemical Industries Ltd. (Osaka, Japan). Concentrated hydrochloric acid was purchased from Nacalai Tesque, Inc. (Kyoto, Japan).

Synthesis of homo- and copolymers of poly{N-[3-(dimethylamino)propyl](meth)acrylamide hydrochloride} (P3-P12)

While the following general procedure was performed for all the polymers containing N-[3-(dimethylamino)propyl](meth)acrylamide hydrochloride, different concentrations and volumes of ingredients were used depending on the types of polymers as listed in Supplementary Table 3.

N-[3-(dimethylamino)propyl](meth)acrylamide hydrochloride was first prepared by dropwise addition of concentrated hydrochloric acid to N-[3-(dimethylamino)propyl](meth)acrylamide while being stirred in an ice water bath.

For the homopolymers and binary copolymers, N-[3-(dimethylamino)propyl](meth)acrylamide hydrochloride and distilled water were mixed in a three-necked flask equipped with a stirrer, a thermometer, and a cooling unit. The mixture was then heated to 60°C. For the binary copolymers, acrylamide or acrylic acid was added dropwise. Ammonium persulfate was then gradually added as a radical polymerization initiator. The mixture was kept at 60°C overnight in order for polymerization to proceed.

For the ternary copolymers, N-[3-(dimethylamino)propyl](meth)acrylamide hydrochloride, acrylamide, and acrylic acid and distilled water were all mixed in a three-necked flask equipped with a stirrer, a thermometer, and a cooling unit. The mixture was heated to 60°C, and ammonium persulfate was then gradually added as a radical polymerization initiator. The mixture was kept at 60°C overnight for polymerization to proceed.

Polyacrylamide (P13)

186.59 g of distilled water was stirred in a three-necked flask equipped with a stirrer, a thermometer, and a cooling unit, and it was then heated to 70°C. After dropwise addition of acrylamide (40.0% (w/w), 62.20 g, 0.35 mol), ammonium persulfate (28.5% (w/w), 2.63 g, 0.003 mol) was gradually added as a radical polymerization initiator. The mixture was kept at 70°C overnight for polymerization to proceed and to give an aqueous solution of homopolymer consisting of acrylamide.

Polyacrylic acid (P14)

189.16 g of distilled water was stirred in a three-necked flask equipped with a stirrer, a thermometer, and a cooling unit, and it was then heated to 60°C. After dropwise addition of acrylic acid (40.0% (w/w), 63.06 g, 0.35 mol), ammonium persulfate (28.5% (w/w), 2.63 g, 0.003 mol) was gradually added as a radical polymerization initiator. The mixture was kept at 60°C overnight for polymerization to proceed and to give an aqueous solution of homopolymer consisting of acrylic acid.

Ternary copolymer consisting of diallylamine hydrochloride, acrylamide, and acrylic acid at a 1:1:2 molar ratio (P18)

Diallylamine hydrochloride (65.4% (w/w), 20.43 g, 0.1 mol) and 110.94 g of distilled water were mixed in a four-necked flask equipped with a stirrer, a thermometer, and a cooling unit. The mixture was then heated to 65°C. Ammonium persulfate (28.5% (w/w), 6.41 g, 0.008 mol) was added as a radical polymerization initiator and incubated for 30 min at 65°C. In another glass flask, a mixture of acrylamide (97%, 7.33 g, 0.10 mol), acrylic acid (99%, 14.56 g, 0.20 mol), and distilled water (21.15 g) was prepared. The latter mixture was slowly added to the first mixture dropwise over a period of 3 h. The mixture was kept at 65°C overnight for polymerization to proceed and to give an aqueous solution of copolymer consisting of diallylamine hydrochloride, acrylamide, and acrylic acid.

Binary copolymer consisting of diallylmethylamine hydrochloride and acrylamide at a 8:1 mole ratio (P21)

Diallylmethylamine hydrochloride was first prepared by adding concentrated hydrochloric acid to diallylmethylamine dropwise while being stirred in an iced water bath. Diallylmethylamine hydrochloride (67.79% (w/w), 19.17 g, 0.088 mol), acrylamide (97.0%, 0.81 g, 0.011 mol), and 25.94 g of distilled water were all mixed in a three-necked flask equipped with a stirrer, a thermometer, and a cooling unit. The mixture was then heated to 55°C. Ammonium persulfate (28.5% (w/w), 3.17 g, 0.004 mol) was gradually added as a radical polymerization initiator. The mixture was kept at 55°C overnight for polymerization to proceed and to give an aqueous solution of copolymer consisting of diallylmethylamine hydrochloride and acrylamide.

Supplementary Table 2: Polymers used in this study.

N/Ds: Not disclosed by the supplier.
Abbreviations
Poly(allylamine hydrochloride): P(AA[HCl])
Poly{N-[3-(dimethylamino)propyl](meth)acrylamide} hydrochloride: P(DMAPMA[HCl])
Poly(diallylamine hydrochloride): P(DAA[HCl])
poly(diallylmethylamine hydrochloride): P(DAMA[HCl])
Acrylamide: AAm
Acrylic acid: AAc
Sulfur dioxide: SO_2
Maleic acid: MA
* Ratio of monomers used in synthesis.

Poymer number	Types of repeat units	Chemical structure of the repeat units	Ratio of repeat units*	MW (average)	cationic density [meq/g]	Souces
P1	P(AA[HCl])	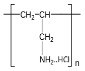	Homopolymer	3,000	17.514	Nittobo Medical`s product
P2	P(AA[HCl]/MA)	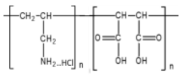	N/Ds	Not determined	7.084	Nittobo Medical`s product
P3	P(DMAPMA[HCl])	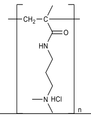	Homopolymer	49771	2.419	Synthesized
P4	P(DMAPMA[HCl]/AAm)	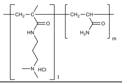	3:1	56,087	4.340	Synthesized
P5			1:1	80,348	3.600	Synthesized
P6			1:3	130,525	2.381	Synthesized
P7	P(DMAPMA[HCl])/AAc)	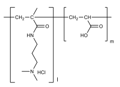	3:1	Not determined	4.436	Synthesized
P8			1:1	Not determined	3.805	Synthesized
P9			1:3	Not determined	2.667	Synthesized
P10	P(DMAPMA[HCl]/AAm/AAc)	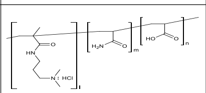	1:1:1	183482	2.995	Synthesized
P11			2:1:1	152234	3.700	Synthesized
P12			1:1:2	132408	2.565	Synthesized
P13	AAm (control)	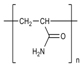	Homopolymer	19597	0.000	Synthesized
P14	AAc (control)	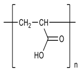	Homopolymer	Not determined	0.000	Synthesized
P15	P(DAA[HCl])	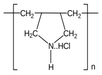	Homopolymer	5,000	10.292	Nittobo Medical`s product
P16	P(DAA[HCl]/SO_2)	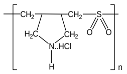	1:1	5,000	5.059	Nittobo Medical`s product
P17	P(DAA[HCl]/MA)	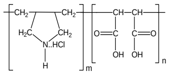	N/Ds	Not determined	4.594	Nittobo Medical`s product
P18	P(DAA[HCl]/AAm/AAc)	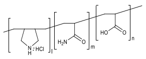	1:1:2	Not determined	3.554	Synthesized
P19	P(DAMA[HCl])	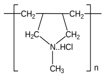	Homopolymer	5,000	6.773	Nittobo Medical`s product
P20	P(DAMA[HCl]/SO_2)	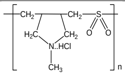	1:1	3,000	4.723	Nittobo Medical`s product
P21	P(DAMA[HCl]/AAm)	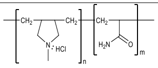	8:1	14685	6.773	Synthesized
P22	P(DAMA[HCl]/MA)	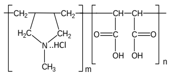	N/Ds	Not determined	5.121	Nittobo Medical`s product

Supplementary Table 3: Composition for the synthesis of poly{N-[3-(dimethylamino)propyl](meth)acrylamide hydrochloride} (P(DMAPMA[HCl]).
* Only the concentration (%) of the DMAPMA[HCl] monomer was varied.

		Amount of ingredient used in synthesis				
**Poymer number**	**Molar ratio of the monomers**	**DMAPMA[HCl]**	**Acrylamide**	**Acrylic acid**	**Ammonium persulfate 28.5% (w/w)**	**Distilled water**
P3	Homopolymer	74.45% (w/w)<br>41.65g (0.15 mol)	0.00	0.00	2.40g (0.003 mol)	268.43g
P4	3:1	73.67% (w/w)<br>42.09g (0.15 mol)	10% (w/w)<br>35.90g (0.05 mol)	0.00	3.20g (0.004 mol)	267.99g
P5	1:1	73.67% (w/w)<br>33.67g (0.12 mol)	10% (w/w)<br>86.16g (0.12 mol)	0.00	3.84g (0.005 mol)	214.39g
P6	1:3	73.67% (w/w)<br>19.64g (0.07 mol)	10% (w/w<br>150.78g (0.21 mol)	0.00	4.48g (0.006 mol)	125.06g
P7	3:1	73.67% (w/w)<br>42.09g (0.15 mol)	0.00	10% (w/w)<br>36.03g (0.05 mol)	3.20g (0.004 mol)	267.99g
P8	1:1	73.67% (w/w<br>33.67g, (0.12 mol)	0.00	10% (w/w)<br>86.47g (0.12 mol)	3.84g (0.005 mol)	214.39g
P9	1:3	73.67% (w/w)<br>19.64g, (0.07 mol)	0.00	10% (w/w)<br>151.33g (0.21 mol)	4.48g (0.006 mol)	125.06g
P10	1:1:1	51.20% (w/w)<br>24.21g, (0.06mol)	97.0%<br>4.40g (0.06 mol)	99.0%<br>4.37g (0.06 mol)	2.88g (0.004 mol)	176.95g
P11	2:1:1	51.20% (w/w)<br>32.30g, (0.08mol)	97.0% (w/w)<br>2.93g (0.04 mol)	99%<br>2.91g (0.04 mol)	2.56g (0.003 mol)	184.49g
P12	1:1:2	51.20% (w/w)<br>16.15g, (0.04mol)	97.0% (w/w)<br>2.93g (0.104 mol)	99%<br>5.82g (0.08 mol)	2.56g (0.003 mol)	143.86g

Supplementary Table 4: Evaluation of product formation for the polymers specifically synthesized for this study.

1) After synthesis, polymers were precipitated by optimum organic solvents. Yields were calculated based on the weights of the precipitated products and total amount of solid material after the evaporation of water, which includes unreacted monomers.

2) The formation of high molecular weight materials was checked by gel permeation chromatography (GPC). Yield was calculated based on peak areas of the polymer and unreacted monomers.

N.P.: Not performed. However, at least either 1) or 2) was performed for all the polymers.

Abbreviations

Poly{N-[3-(dimethylamino)propyl](meth)acrylamide} hydrochloride: P(DMAPMA[HCl])

Poly(diallylamine hydrochloride): P(DAA[HCl])

poly(diallylmethylamine hydrochloride): P(DAMA[HCl])

Acrylamide: AAm

Acrylic acid: AAc

Sulfur dioxide: SO_2

Maleic acid: MA

* Ratio of monomers used in synthesis

			1) Precipiation followed by yield evaluation		2) GPC analysis	
Poymer number	Types of repeat units	Ratio of repeat units*	Organic solvents	Yield (%)	Measured MW	Yield (%)
P3	P(DMAPMA[HCl])	Homopolymer	Isopropylalcohol	99.24	49771	96.18
P4	P(DMAPMA[HCl]/AAm)	3:1	Isopropylalcohol	106.6	56087	99.64
P5		1:1	Isopropylalcohol	113.74	80348	100
P6		1:3	Isopropylalcohol	133.1	130525	100
P7	P(DMAPMA[HCl]/AAc)	3:1	Isopropylalcohol	102.34	N.P.	N.P.
P8		1:1	Isopropylalcohol	98.06	N.P.	N.P.
P9		1:3	Isopropylalcohol NaOH	89.16	N.P.	N.P.
P10	P(DMAPMA[HCl]/AAm/AAc)	1:1:1	N.P.	N.P.	183482	98.38
P11		2:1:1	N.P.	N.P.	152234	98.74
P12		1:1:2	N.P.	N.P.	132408	99.72
P13	AAm (control)	Homopolymer	Isopropylalcohol	129.58	19597	100
P14	AAc (control)	Homopolymer	Isopropylalcohol & NaOH	99.85	N.P.	N.P.
P18	P(DAA[HCl]/AAm/AAc)	1:1:2	Acetone	118.05	N.P.	N.P.
P21	P(DAMA[HCl]/AAm)	8:1	N.P.	N.P.	14685	94.67
